# Autoimmune diseases and the risk and prognosis of latent autoimmune diabetes in adults

**DOI:** 10.1007/s00125-024-06303-4

**Published:** 2024-10-28

**Authors:** Cornelia Santoso, Yuxia Wei, Emma Ahlqvist, Tiinamaija Tuomi, Sofia Carlsson

**Affiliations:** 1 Institute of Environmental Medicine, Karolinska Institutet, Stockholm, Sweden; 2 Department of Clinical Sciences in Malmö, Clinical Research Centre, Lund University, Malmö, Sweden; 3 Division of Endocrinology, Abdominal Center, Helsinki University Hospital, Helsinki, Finland; 4 Institute for Molecular Medicine Finland, University of Helsinki, Helsinki, Finland; 5 Folkhälsan Research Center, Helsinki, Finland; 6 Research Program for Clinical and Molecular Medicine, University of Helsinki, Helsinki, Finland

**Keywords:** Autoimmune disease, Diabetic retinopathy, Epidemiology, Latent autoimmune diabetes in adults, Prognosis, Risk factor

## Abstract

**Aims/hypothesis:**

The aim of this study was to clarify the impact of autoimmune disease (AD) comorbidity on the risk and prognosis of latent autoimmune diabetes in adults (LADA).

**Methods:**

We used data from a Swedish study comprising newly diagnosed cases of LADA (*n*=586, stratified into LADA^low^ and LADA^high^ by autoantibody levels), type 2 diabetes (*n*=2003) and matched control participants (*n*=2355). Information on 33 ADs and diabetic retinopathy was obtained by linkage to regional and national registers. We estimated the ORs for LADA and type 2 diabetes in relation to ADs before diabetes diagnosis, and the HRs for diabetic retinopathy after diabetes diagnosis. We performed functional pathway analyses to explore biological mechanisms driving the associations.

**Results:**

Individuals with ADs exhibit an increased susceptibility to LADA (OR 1.70; 95% CI 1.36, 2.13), particularly those with thyroid dysfunction (OR 1.88; 95% CI 1.38, 2.56), inflammatory bowel disease (OR 1.78; 95% CI 1.00, 3.16) or vitiligo (OR 3.91; 95% CI 1.93, 7.94), with stronger associations being observed for the LADA^high^ phenotype. Only psoriasis was linked to type 2 diabetes (OR 1.47; 95% CI 1.08, 1.99). The biological pathways shared by LADA and ADs revolved around immune responses, including innate and adaptive immune pathways. The HRs for diabetic retinopathy in LADA patients with and without AD vs those with type 2 diabetes were 2.11 (95% CI 1.34, 3.32) and 1.68 (95% CI 1.15, 2.45), respectively.

**Conclusions/interpretation:**

We confirm that several common ADs confer an excess risk of LADA, especially LADA with higher GADA levels, but having such a comorbidity does not appear to affect the risk of diabetic retinopathy.

**Graphical Abstract:**

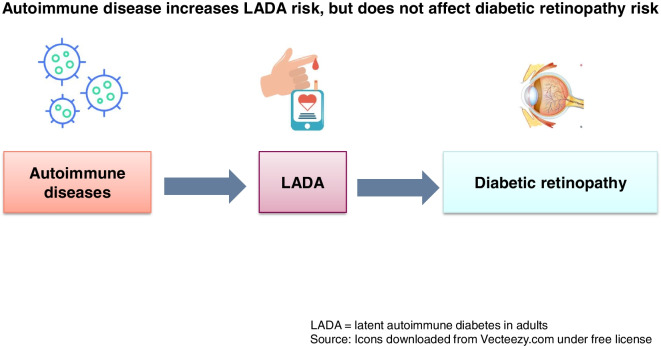

**Supplementary Information:**

The online version of this article (10.1007/s00125-024-06303-4) contains peer-reviewed but unedited supplementary material.


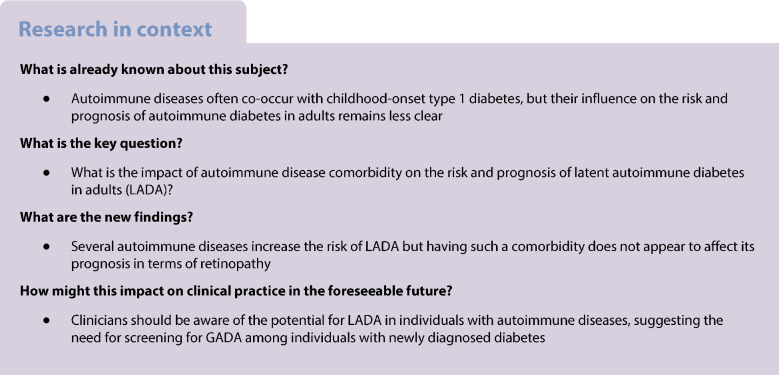


## Introduction

Autoimmune diseases (ADs) are known to cluster, such so that individuals with one AD are at an increased risk of developing other ADs [1]. It is believed that shared genetic susceptibility and environmental triggers, leading to dysregulation of the immune system, contribute to this phenomenon [1]. A higher prevalence of several ADs, including thyroid dysfunction, coeliac disease, Addison’s disease, inflammatory bowel disease, vitiligo and rheumatoid arthritis has been observed in patients with type 1 diabetes with childhood diagnosis [1, 2]. Much less is known about co-aggregation of ADs and autoimmune diabetes that is diagnosed during adulthood.

Latent autoimmune diabetes in adults (LADA) is the most common form of autoimmune diabetes with adult onset, accounting for 3–14% of all diabetes in adults [3]. People with LADA tend to be positive for one autoantibody (GADA) rather than multiple autoantibodies, as observed in children with type 1 diabetes; a smaller proportion have the high-risk HLA DR-DQ haplotypes, and insulin deficiency is less pronounced [4]. Only a few small-scale, cross-sectional studies have investigated AD comorbidity in LADA [5–10]. They report higher prevalence of thyroid dysfunction, Addison’s disease or coeliac disease autoantibodies when comparing LADA with type 2 diabetes [5–10] or diabetes-free control participants [6]. Whether other ADs are over-represented in LADA is not clear, and the influence of family history of ADs on LADA risk also remains to be investigated.

People with LADA are at increased risk of vascular disease, including diabetic retinopathy [11]. Most ADs also confer excess risk of vascular disease [12]. Having AD comorbidity may thus add to the already high risk of diabetic retinopathy in LADA patients. In childhood-onset type 1 diabetes, individuals with ADs have been shown to have elevated HbA_1c_ levels [13] and increased prevalence of ischaemic heart disease [14], renal failure [15] and retinopathy [16]. However, a recent Swedish study found no differences in HbA_1c_ levels [17]. In LADA, a positive association between thyroid dysfunction and HbA_1c_ levels was noted [18], but to the best of our knowledge, no previous study has assessed the prognosis of LADA patients in relation to AD comorbidity.

Our aim was to clarify how ADs impact the risk and prognosis of LADA using data from a Swedish study featuring almost 600 LADA patients and 2000 type 2 diabetes patients. We examined the risk of LADA and type 2 diabetes in relation to 33 ADs and family history of ADs, and estimated the incidence of diabetic retinopathy in LADA with and without AD. We also explored the biological mechanisms using post-genome-wide association studies (GWAS) data.

## Methods

### Study population

We used data from the Epidemiological Study of Risk Factors for LADA and Type 2 Diabetes (ESTRID), nested within the ANDIS (All New Diabetics in Scania) biobank and incidence register covering the Swedish region of Scania [19]. Since 2010, all incident cases of LADA (termed ‘slowly evolving, immune-mediated diabetes of adults’ by WHO [20]) in ANDIS have been invited to participate in ESTRID, together with randomly sampled type 2 diabetes patients (ratio 1:4) from the ANDIS biobank and diabetes-free control participants (ratio 1:6) randomly sampled from the population of Scania and matched by participation date and residential area [21]. All cases and control participants recruited between 2010 and 2019 were eligible for the present study (LADA, *n*=586; type 2 diabetes, *n*=2003; control participants, *n*=2355). The Swedish Ethical Review Board approved the study (numbers 2010/336-31/1 and 2018/1036-32).

### Diabetes classification

Cases of diabetes were diagnosed in the healthcare system of Scania. Patients aged ≥35 years at diagnosis were classified as having LADA if they were GADA-positive (≥10 U/ml) with C-peptide ≥0.3 nmol/l (measured using a Cobas e601 analyser; Roche Diagnostics, Germany) or ≥0.2 nmol/l (measured using an IMMULITE 2000 assay system; Siemens Healthcare, UK) and as having type 2 diabetes if they were GADA-negative with C-peptide ≥0.72 nmol/l (Cobas) or ≥0.60 nmol/l (IMMULITE). LADA patients were further categorised into LADA^high^ (GADA ≥250 U/ml) and LADA^low^ (GADA <250 U/ml) based on the median GADA level. The sensitivity and specificity of GADA measurement using ELISA (RSR, UK) were 0.84 and 0.98, respectively. HOMA-IR and HOMA-B values were calculated from fasting glucose and C-peptide levels. Genotyping was conducted using iPlex (Sequenom, USA) or TaqMan assays (Thermo Fisher Scientific, USA) [19]. We used three SNPs (rs3104413, rs2854275 and rs9273363) to predict HLA-DRB1 (DR3/DR4) and HLA-DQB1 (DQ2/DQ8) genotypes. An overall accuracy of 99.3% has been demonstrated for this approach [22].

### Autoimmune diseases

**Fig. 1:**
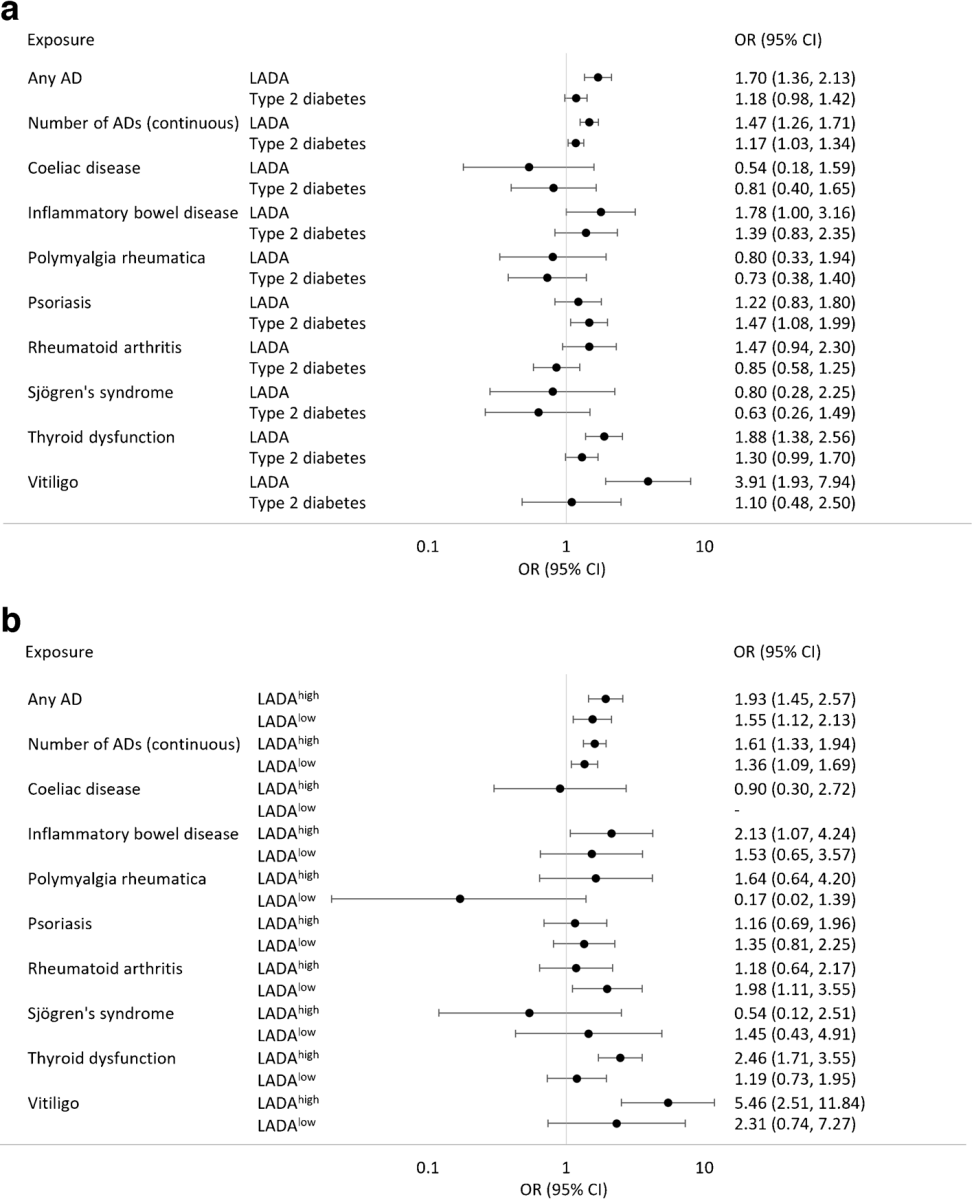
(**a**) Risk of LADA and type 2 diabetes for various ADs, and (**b**) risk of LADA with high (LADA^high^) and low (LADA^low^) GADA levels for various ADs. ORs and 95% CIs were adjusted for age, sex, education, smoking, physical activity, BMI, family history of type 1 diabetes, family history of type 2 diabetes and family history of any AD. ORs for individual ADs were mutually adjusted for the other ADs

Information on ADs was obtained through the National Patient Register (NPR) (1997–2019) and the Scania Healthcare Register (2004–2019) (see electronic supplementary material [ESM] Fig. 1). The NPR provides nationwide coverage of inpatient care since 1987 and outpatient care since 2001 [23] and the Scania register provides almost complete coverage of primary care since 2004 [24]. We retrieved information on diagnoses of 33 ADs (ESM Table 1) that have been shown to be relatively prevalent in Sweden [25]. In addition, we used self-reported information on diagnoses of coeliac disease, thyroid dysfunction, Sjögren’s syndrome, systemic lupus erythematosus, ulcerative colitis or Crohn’s disease, vitiligo, psoriasis, multiple sclerosis or rheumatoid arthritis and year of diagnosis. In the analyses, we integrated self-reported information with the register data and combined diagnoses of Graves’ disease and Hashimoto’s disease into thyroid dysfunction, and diagnoses of ulcerative colitis and Crohn’s disease into inflammatory bowel disease. There were also questions on family history of these ADs in the patient’s mother, father, siblings or other relatives.

### Diabetic retinopathy

Information on diabetic retinopathy (hereafter referred to as ‘retinopathy’) was retrieved from the NPR, the Cause-of-Death Register (CDR) and the National Diabetes Register (NDR) [26]. Retinopathy was assessed in the patients with diabetes, and was defined as the first occurrence in NPR or NDR of severe non-proliferative retinopathy, pre-proliferative diabetic retinopathy, proliferative diabetic retinopathy, diabetes with advanced eye disease, other proliferative retinopathy, diabetic cataract, retinal haemorrhage, visual impairment or vitreous haemorrhage, or death attributable to diabetic retinopathy in the CDR. The ICD-10 codes (https://icd.who.int/browse10/2019/en) for these forms of retinopathy are given in ESM Table 2.

### Covariates

Information on covariates was collected by questionnaire at enrolment. BMI was based on self-reported weight and height. Smoking history was used to categorise individuals into never, former or current smokers. Based on validated questions [27], individuals were categorised as sedentary or displaying light, moderate or high physical activity. Family history of type 1 diabetes in first-degree relatives (mother, father, siblings or children) or grandparents was defined as diagnosis at age <40 years with insulin treatment; otherwise, the diagnosis was recorded as type 2 diabetes. Educational level was categorised into primary, secondary or tertiary. We used median values to impute missing data on lifestyle covariates (missing rate 1.5%), and created a dummy variable to indicate missingness, which we incorporated into regression models. Information on HbA_1c_ levels and diabetes medications was retrieved from NDR and the prescribed drug register (2005–2019), respectively.

### Statistical analyses

We assessed differences in the distribution of baseline characteristics using the *χ*^2^ test (for proportions with expected frequency ≥5), Fisher’s exact test (for proportions with expected frequency <5), Student’s *t* test (unpaired) for means and the Wilcoxon test for medians. Values were considered significant at *p*<0.05. R version 4.3.3 (R Foundation for Statistical Computing, Austria), SAS 9.4 (IBM, USA) and STATA 17.0 (StataCorp, USA) were used.

#### Case–control analyses

Conditional logistic regression was used to estimate ORs and 95% CIs for LADA/type 2 diabetes in relation to ADs and family history of ADs in first-degree relatives. We used information on ADs occurring at least one year prior to the index date (date of diagnosis for patients or date of enrolment for control participants). ORs were calculated in relation to any vs no AD, number of ADs, and individual ADs if there were five or more affected LADA patients. Regarding family history, ORs were estimated in relation to any AD, number of ADs, number of affected relatives and individual ADs. Model 1 was adjusted for age and sex; model 2 was additionally adjusted for education, smoking, physical activity and BMI; and model 3 was additionally adjusted for family history of type 1 diabetes, type 2 diabetes and any AD. For analyses of individual ADs, the models were mutually adjusted for other ADs. The results of model 3 are presented below unless otherwise stated. We calculated the attributable proportion due to interaction [28] to assess additive interaction between having an AD and having first-degree relatives with AD.

#### Cohort analyses

Cox proportional hazard regression models, with age as the time scale, were used to estimate HRs and 95% CIs for retinopathy in LADA with and without AD, compared with type 2 diabetes. Person-years were counted from baseline date until date of the first event or end of follow-up (31 December 2019). The main model was adjusted for sex, calendar year at diabetes diagnosis, diabetes duration, education, smoking, alcohol, physical activity, BMI, HbA_1c_, BP, lipids and eGFR. HbA_1c_ trajectories for LADA with and without AD were estimated using generalised linear models adjusted for age, sex and calendar year.

### Sensitivity analysis

We performed analyses restricted to register information on ADs, and adjusted the analyses of individual ADs and family history of ADs for multiple testing. As ADs were more common in female participants than male participants, we stratified the analyses by sex. As a post hoc analysis, we estimated the risk of type 2 diabetes in relation to ADs while excluding psoriasis. We analysed retinopathy in LADA vs type 2 diabetes while adjusting for glucose-lowering drugs, statins and antihypertensive treatment, with non-diabetic retinopathy death as a competing event using the Fine–Gray method [29].

### Biological pathway analysis

For ADs associated with LADA, we explored the underlying biology through pathway analysis using FUMA, a web-based platform that incorporates various biological resources to functionally annotate GWAS results and prioritise genes [30]. First, we used the SNP2GENE function to apply positional mapping (restricted to exonic and splicing SNPs with a combined annotation-dependent depletion [CADD] score ≥12.37), expression quantitative trait loci (eQTL) mapping (performed using GTEx version 8 eQTLs with false discovery rate [FDR] <0.05), and chromatin interaction mapping (performed using Hi-C data, with interactions filtered by FDR <1×10^−6^) to GWAS summary statistics for LADA [31], Crohn’s disease [32], ulcerative colitis [32], Graves’ disease [32], hypothyroidism [33] and vitiligo [34]. Subsequently, we chose genes present in both LADA and the respective ADs. Finally, we used the GENE2FUNC function to annotate the shared genes for biological mechanisms. Details of the mechanisms are shown in ESM Fig. 2.

## Results

### Characteristics

The prevalence of AD was 34% in LADA patients, 26% in those with type 2 diabetes, and 22% in control participants, with thyroid dysfunction being most prevalent, followed by psoriasis and rheumatoid arthritis (Table 1 and ESM Table 3). LADA patients had lower HOMA-B, HOMA-IR and C-peptide and a higher prevalence of high-risk HLA genotypes and family history of type 1 diabetes and ADs than type 2 diabetes patients. The prevalence of ADs (38% vs 28%), family history of AD (61% vs 48%) and high-risk HLA genotypes (67% vs 53%) was higher in LADA^high^ vs LADA^low^ patients, while C-peptide, HOMA-B and HOMA-IR were lower (Table 1).

**Table 1:** Baseline characteristics of the study population

	Control participants (*n*=2355)	LADA (*n*=586)	Type 2 diabetes (*n*=2003)	*p*	LADA^low^ (*n*=277)	LADA^high^ (*n*=298)	*p*
Age (years)	58.8±13.8	59.0±12.4	63.1±10.4	<0.001	59.6 (12.3)	58.4 (12.4)	0.246
Female	1243 (52.8)	274 (46.8)	792 (39.5)	0.002	116 (41.9)	154 (51.7)	0.019
BMI	26.0±4.2	28.4±5.6	31.1±5.3	<0.001	29.1 (6.0)	27.6 (5.1)	0.001
BMI ≥25 kg/m^2^	1280 (54.4)	417 (71.2)	1853 (92.5)	<0.001	213 (76.9)	196 (65.8)	0.003
Family history of diabetes	1002 (42.5)	373 (63.7)	1306 (65.2)	0.489	168 (60.6)	196 (65.8)	0.203
Family history of type 1 diabetes	77 (3.3)	66 (11.3)	111 (5.5)	<0.001	27 (9.7)	37 (12.4)	0.309
Family history of type 2 diabetes	775 (32.9)	280 (47.8)	1128 (56.3)	<0.001	128 (46.2)	146 (49.0)	0.504
GADA	NA	250.0 (31.5–250.0)	NA		30 (17–83)	250 (250–250)	<0.001
C-peptide	NA	0.71 (0.45–1.20)	1.20 (0.96–1.60)	<0.001	0.9 (0.5–1.3)	0.6 (0.4–0.9)	<0.001
HOMA-B	NA	40.5 (14.7–69.0)	70.9 (44.3–95.8)	<0.001	46.8 (19.6–81.6)	30.9 (13.0–59.0)	<0.001
HOMA-IR	NA	2.82 (1.81–4.47)	3.56 (2.72–4.76)	<0.001	3.2 (2.2–4.8)	2.5 (1.7–4.1)	0.002
HbA_1c_ (mmol/mol)	NA	57.0 (47.0–82.0)	50.0 (44.0–63.0)	<0.001	56.0 (47.0–79.0)	60.0 (49.0–87.0)	0.075
HbA_1c_ (%)	NA	7.4 (6.5–9.7)	6.7 (6.2–7.9)	<0.001	7.3 (6.5–9.4)	7.6 (6.6–10.1)	0.075
High-risk HLA genotypes	NA	238 (59.9)	395 (31.2)	<0.001	107 (53.2)	131 (66.8)	0.006
Family history of AD	1182 (50.2)	321 (54.8)	910 (45.4)	<0.001	134 (48.4)	183 (61.4)	0.002
Concomitant AD							
Any	512 (21.7)	198 (33.8)	517 (25.8)	<0.001	78 (28.2)	114 (38.3)	0.010
Coeliac disease	37 (1.6)	6 (1.0)	22 (1.1)	0.878	2 (0.7)	4 (1.3)	0.687
Inflammatory bowel disease	48 (2.0)	21 (3.6)	43 (2.1)	0.049	8 (2.9)	13 (4.4)	0.346
Polymyalgia rheumatica	28 (1.2)	10 (1.7)	39 (1.9)	0.707	2 (0.7)	8 (2.7)	0.109
Psoriasis	133 (5.6)	44 (7.5)	172 (8.6)	0.406	21 (7.6)	21 (7.0)	0.806
Rheumatoid arthritis	90 (3.8)	38 (6.5)	90 (4.5)	0.050	20 (7.2)	17 (5.7)	0.459
Sjögren’s syndrome	21 (0.9)	7 (1.2)	15 (0.7)	0.308	4 (1.4)	3 (1.0)	0.716
Thyroid dysfunction	203 (8.6)	104 (17.7)	216 (10.8)	<0.001	33 (11.9)	66 (22.1)	0.001
Vitiligo	21 (0.9)	19 (3.2)	19 (0.9)	<0.001	4 (1.4)	15 (5.0)	0.016

Values are means ± SD, *n* (%) or median (IQR)

The percentages shown are calculated for participants without missing data

Family history of type 1 or type 2 diabetes included parents, siblings, children or grandparents with the condition, while family history of diabetes also included other relatives. Individuals carrying HLA genotypes DR3/3, DR3/4 or DR4/4 or haplotypes of DR4-DQ8 or DR3-DQ2 were considered to have a high genetic risk of LADA or type 1 diabetes. Those with HLA genotypes DR3/X, DR4/X or DRX/X (where X is neither 3 nor 4) and DR4-DQ7 were classified as having low or intermediate risk. The *p* values are for LADA vs type 2 diabetes and LADA^high^ vs LADA^low^

NA, not applicable

### AD and the risk of LADA and type 2 diabetes

Individuals with any AD (OR 1.70; 95% CI 1.36, 2.13) were more likely to develop LADA than those without, and the risk increased with the number of ADs (OR 1.47; 95% CI 1.26, 1.71 per additional AD) (Fig. 1 and ESM Table 4). Of the individual ADs, excess risk of LADA was observed in relation to thyroid dysfunction (OR 1.88; 95% CI 1.38, 2.56), inflammatory bowel disease (OR 1.78; 95% CI 1.00, 3.16) and vitiligo (OR 3.91; 95% CI 1.93, 7.94) (Fig. 1 and ESM Table 4). After adjusting for multiple testing, the association with inflammatory bowel disease was no longer significant (ESM Table 5). The association between LADA and thyroid dysfunction was observed for hyperthyroidism (Graves’ disease: OR 2.35; 95% CI 1.12, 4.95) and hypothyroidism (Hashimoto’s disease: OR 1.66; 95% CI 1.12, 2.47) (ESM Table 6). Excess risk of LADA in relation to any AD, thyroid dysfunction and vitiligo was observed both in male and female participants (ESM Fig. 3). Stratifying the analyses by GADA levels indicated that associations with ADs in general were stronger for LADA^high^ (OR 1.93; 95% CI 1.45, 2.57 for any vs no AD) than for LADA^low^ (OR 1.55; 95% CI 1.12, 2.13 for any vs no AD) (Fig. 1 and ESM Table 7). These associations remained after adjustment for lifestyle factors and family history of diabetes and ADs (ESM Table 7). The results were similar in sensitivity analyses based exclusively on diagnoses in the patient registers (ESM Tables 8 and 9). There was an association between the number of ADs and type 2 diabetes (OR 1.17; 95% CI 1.03, 1.34) (Fig. 1 and ESM Table 4), which was driven by the excess risk conferred by psoriasis (OR 1.47; 95% CI 1.08, 1.99). Once psoriasis was excluded, ADs were not associated with type 2 diabetes (ESM Table 10).

Increased risk of LADA was seen in individuals with at least two ADs in first-degree relatives (OR 1.54; 95% CI 1.10, 2.14) or two affected relatives (OR 1.57; 95% CI 1.09, 2.26). Similar risks were observed for LADA^high^ patients, but there was no excess risk for LADA^low^ patients or those with type 2 diabetes (Table 2). Regarding individual ADs in relatives, vitiligo was associated with LADA (OR 3.45; 95% CI 2.02, 5.89), including both LADA^low^ and LADA^high^ patients, while multiple sclerosis (OR 2.44; 95% CI 1.10, 5.40) and thyroid dysfunction (OR 1.45; 95% CI 1.02, 2.06) were associated with LADA^high^ (ESM Tables 11 and 12). However, after adjusting for multiple testing, only the associations between vitiligo and LADA and between vitiligo and LADA^high^ remained significant (ESM Table 13). We observed no interactions between having any AD and having first-degree relatives with AD on the risk of LADA or type 2 diabetes (ESM Table 14).

**Table 2:** Family history of AD in first-degree relatives and risk of LADA, LADA^high^, LADA^low^ and type 2 diabetes

	LADA		LADA ^high^		LADA ^low^		Type 2 diabetes	
	Number of cases/control participants	OR (95% CI)	Number of cases/control participants	OR (95% CI)	Number of cases/control participants	OR (95% CI)	Number of cases/control participants	OR (95% CI)
Family history of any AD								
No	361/1574	Reference	164/1574	Reference	190/1574	Reference	1367/1574	Reference
Yes	225/781	1.08 (0.88, 1.32)	134/781	1.34 (1.03, 1.74)	87/781	0.85 (0.64, 1.14)	636/781	0.90 (0.76, 1.06)
Number of ADs in the family								
0	361/1574	Reference	164/1574	Reference	190/1574	Reference	1367/1574	Reference
1	156/618	0.96 (0.76, 1.20)	88/618	1.14 (0.85, 1.53)	66/618	0.81 (0.59, 1.12)	503/618	0.88 (0.73, 1.05)
2+	69/163	1.54 (1.10, 2.14)	46/163	2.08 (1.40, 3.09)	21/163	1.00 (0.60, 1.67)	133/163	0.98 (0.73, 1.33)
Continuous		1.14 (1.00, 1.31)		1.32 (1.12, 1.56)		0.95 (0.77, 1.17)		0.95 (0.85, 1.07)
Number of relatives with any AD								
0	361/1574	Reference	164/1574	Reference	190/1574	Reference	1367/1574	Reference
1	168/649	0.98 (0.78, 1.22)	93/649	1.15 (0.87, 1.54)	72/649	0.85 (0.62, 1.16)	526/649	0.89 (0.75, 1.06)
2+	57/132	1.57 (1.09, 2.26)	41/132	2.26 (1.48, 3.46)	15/132	0.87 (0.48, 1.57)	110/132	0.93 (0.66, 1.31)
Continuous		1.14 (0.98, 1.33)		1.37 (1.13, 1.65)		0.90 (0.71, 1.13)		0.94 (0.83, 1.07)

ORs and 95% CIs were adjusted for age, sex, education, smoking, physical activity, BMI, having any ADs at baseline, type 1 diabetes in first-degree relatives and type 2 diabetes in first-degree relatives

Individuals with LADA and AD were significantly older, more likely to be female, and had higher levels of GADA and a higher prevalence of family history of ADs and retinopathy, but a lower prevalence of overweight/obesity, than those without concomitant AD. There were no differences in the prevalence of family history of diabetes, high-risk HLA genotypes, education, smoking, baseline HbA_1c_, or the proportions treated with insulin or a combination of insulin and other glucose-lowering drugs (ESM Table 15).

### Biological pathway analyses

The biological pathways linking LADA to Crohn’s disease, ulcerative colitis, hypothyroidism, Graves’ disease and vitiligo are shown in ESM Figs 4–8. A total of 41, 60, 148, 54 and 141 genes were involved in 61, 116, 101, 75 and 113 biological processes for Crohn’s disease, ulcerative colitis, hypothyroidism, Graves’ disease and vitiligo, respectively. The biological pathways linking LADA to the various individual ADs are similar, revolving around immune responses, including both innate and adaptive immune pathways. These pathways involve various types of immune-related cells, such as B cells, T cells and natural killer cells, and diverse immune molecules, including cytokines, immunoglobulins and interferons. The strongest enrichment signals were in the antigen processing and presentation categories, involving various HLA variants (e.g. HLA-DRA) as well as the genes *TAP1*, *TAP2*, *TAPBP*, *MICA*, *MICB* and *PSMB8*. Additionally, excluding MHC regions showed no pathways linking LADA to Crohn’s disease, ulcerative colitis or Graves’ disease, with only a limited number identified for hypothyroidism and vitiligo (ESM Figs 9 and 10), suggesting that these pathways are mostly driven by the MHC.

### ADs and diabetic retinopathy

During the follow-up period (median 5.9 years), we identified 198 retinopathy events, 82 of which occurred in LADA patients. The risk of retinopathy was higher in LADA than in type 2 diabetes, and excess risk was seen in LADA with ADs (HR 2.11; 95% CI 1.34, 3.32) and without AD (HR 1.68; 95% CI 1.15, 2.45) (Table 3). The results were attenuated after adjusting for use of glucose-lowering drugs, statins and antihypertensive drugs (ESM Table 16). During follow-up, a larger proportion of LADA patients with ADs failed to reach the HbA_1c_ target of <53 mmol/mol compared with LADA patients without AD (ESM Fig. 11).

**Table 3:** Incidence of diabetic retinopathy in patients with LADA with and without autoimmune disease comorbidity, compared with type 2 diabetes

	Number of events	Person-years	HR (95% CI)	
			Model 1	Model 2
Type 2 diabetes	116	10,508	Reference	Reference
LADA overall	82	3209	2.32 (1.73, 3.11)	1.81 (1.29, 2.54)
LADA without AD	53	2160	2.20 (1.58, 3.08)	1.68 (1.15, 2.45)
LADA with AD	29	1049	2.56 (1.69, 3.87)	2.11 (1.34, 3.32)

Model 1 was a Cox proportional hazard regression model with attained age as the time scale, adjusted for sex, calendar year at diabetes diagnosis and diabetes duration. Model 2 was additionally adjusted for education, smoking, alcohol consumption (based on amount and frequency in the past year), physical activity, BMI, HbA_1c_, BP, lipids and eGFR. The study population for the analyses comprised people who were free of diabetic retinopathy at baseline

## Discussion

### Main findings

To the best of our knowledge, this is the largest study to date to investigate AD comorbidity in LADA and the first to assess the prognosis of LADA in relation to such diseases. We confirm an elevated risk of LADA in relation to several organ-specific ADs, namely thyroid dysfunction, inflammatory bowel disease and vitiligo. We also found that the risk of LADA increased in tandem with the number of ADs present. We can confirm that a greater proportion of women experience ADs. Nevertheless, in both male and female participants, having an AD was linked to an increased risk of LADA. AD comorbidity did not seem to influence LADA prognosis in terms of retinopathy.

### Main findings in relation to previous studies

The observed link between thyroid dysfunction and LADA confirms previous cross-sectional observations [5–9]. We also showed that excess risk of LADA is conferred by both hyperthyroidism and hypothyroidism. The higher risk of LADA observed in relation to inflammatory bowel disease and vitiligo was novel, but aligns with observations regarding childhood type 1 diabetes [1]. Vitiligo conferred the highest LADA risk, increasing it fourfold. We also noted an increased risk of LADA in individuals with family history of vitiligo. In a recent UK study of childhood-onset type 1 diabetes, involving 22 million individuals and covering 19 ADs, the strongest associations were seen for coeliac disease, Addison’s disease, thyroid disfunction and rheumatoid arthritis; these were associated with three- to more than tenfold increased risks [1]. In general, the associations that we observed between ADs and LADA risk were weaker, which is to be expected as the pathogenesis of LADA includes a milder autoimmune process, coupled with some degree of insulin resistance [4]. Considering this, it is not surprising that, upon stratifying the LADA patients by GADA levels, we and others [8] found stronger associations between ADs and LADA with higher GADA levels. Coeliac disease did not confer an increased risk of LADA, which is consistent with most previous LADA studies [6, 10, 35, 36] but not all [7, 8]. The link between coeliac disease and type 1 diabetes has been attributed to shared genetic effects, but shared environmental risk factors, specifically exposures during early life, such as age at introduction to gluten and virus infections, have also been implicated [37]. Even though the genetic risk factors for LADA and type 1 diabetes overlap, the role of environmental risk factors may differ, especially for early life factors, which may be more important for autoimmune diabetes with onset during childhood rather than adulthood. Whether coeliac disease is associated with type 1 diabetes with adult onset remains to be investigated. Regarding Sjögren’s syndrome, we found no association with LADA, consistent with previous findings in childhood-onset type 1 diabetes [1]. Unfortunately, there were not enough cases to investigate Addison’s disease in relation to LADA risk.

Only psoriasis was associated with type 2 diabetes, confirming previous observations [38, 39]. Shared genetics involved in immune regulation and inflammatory responses [40], including regulation of NF-κB expression via tumour necrosis factor receptor-associated factor 6 (TRAF6), has been suggested as a potential underlying mechanism [39]. In contrast, a previous Swedish study found associations between a range of ADs and type 2 diabetes, including thyroid dysfunction, inflammatory bowel disease and Addison’s disease [38]. Our study may be underpowered to detect such associations. It is also possible that undiagnosed LADA contributed to the associations observed in the former study, as there was no information on GADA positivity.

### Mechanisms

The association between ADs may reflect overlapping genetic and/or environmental risk factors. A Swedish twin study indicated that the co-aggregation of type 1 diabetes with other ADs largely is attributable to shared genetic factors [41]. Notably, we observed associations between LADA and ADs after adjusting for several potentially shared environmental risk factors, such as smoking and obesity, supporting the role of genetic factors. Our finding that family history of ADs was associated with LADA^high^ also supports the existence of shared genetics. Genes in the HLA region are associated with most ADs, but some variants may increase the risk of one AD while providing protection against another [42]. This may explain why there was no difference in the prevalence of high-risk variants of HLA-DRB1 and HLA-DQB1 genotypes between LADA individuals with and without AD, even though these variants have also been linked to increased risk of coeliac disease, thyroid dysfunction and rheumatoid arthritis [43, 44]. However, it is well-known that ADs also share non-HLA loci [45], and this may also contribute to the association between LADA and other ADs. Our pathway analysis revealed that immune responses, encompassing both innate and adaptive pathways, driven by the MHC, were the predominant shared biological pathways between LADA and Crohn’s disease, ulcerative colitis, hypothyroidism, hyperthyroidism and vitiligo. These pathways involve diverse immune cells (B cells, T cells and natural killer cells) and molecules (cytokines, immunoglobulins and interferons).

As noted previously in this cohort [11], LADA was associated with higher incidence of retinopathy compared with type 2 diabetes, largely confirming the results of a previous report [46]. There was no indication that having AD comorbidity was associated with worse LADA prognosis in terms of retinopathy. This contrasts with findings in type 1 diabetes [47, 48] and type 2 diabetes [49]. As observed in previous studies [9, 46, 50], we confirm that individuals with LADA have higher HbA_1c_ than those with type 2 diabetes, but noted no differences in baseline HbA_1c_ levels between LADA with and without concomitant ADs. We hypothesised that the similarity in prognosis may be because individuals with LADA are subject to more diligent monitoring, potentially mitigating adverse effects of the additional ADs. There were no clear differences in the proportion treated with insulin or other glucose-lowering drugs. However, we did not have information on healthcare visits or self-monitoring, which may also contribute to better prognosis. We noted that, during follow-up, a smaller proportion of LADA patients with AD vs those without AD comorbidity reached the HbA_1c_ target. Whether not reaching the HbA_1c_ target eventually manifests as a higher risk of vascular disease remains to be confirmed in future studies with longer follow-up. The limited number of events and rather short follow-up period in our study may not fully capture the consequences of AD comorbidity. Further investigations of the role of AD comorbidity in LADA prognosis, including cardiovascular outcomes, are clearly needed.

### Strengths and limitations

The AD information was drawn from the Swedish Patient Register, which has a high validity of diagnosis (positive predictive values for ADs 74–96%) and extensive coverage [23], and a regional primary care register. Information from the NPR data has been available since 1997, and that from the primary care register has been available since 2004. This implies that ADs diagnosed earlier, including during childhood, will not be detected if the patients had not sought medical care since the data started to be collected. For that reason, we included self-reported AD information in the main analyses. This approach may introduce recall bias, but sensitivity analyses based exclusively on register-based diagnoses largely supported our findings. Our study encompassed a period of up to 22 years prior to LADA diagnosis, and we only included ADs occurring 1 year before the index date in the case–control study to address temporality and avoid surveillance bias. Nevertheless, individuals with ADs may have more frequent healthcare contacts, leading to earlier detection of diabetes and hence overestimation of associations. The small numbers of patients with some of the ADs hampered several of the analyses, and precluded us from investigating associations between LADA and some ADs previously linked to type 1 diabetes [1]. Furthermore, there may be the potential for underdiagnosis of ADs as we lacked data on specific autoimmunity markers. The ADs that we considered included autoimmune, autoinflammatory and chronic inflammatory conditions as they lack precise boundaries [25]. The less than perfect specificity (98%) of the GADA assays implies that some type 2 diabetes patients may be misclassified as having LADA. This would lead to attenuation of the associations between ADs and LADA. In addition, we could not apply a stricter definition of diabetic retinopathy as there are no specific ICD-10 codes for laser therapy or vitreous injections. Finally, the generalisability of our findings to other populations with different healthcare systems remains uncertain.

Our study suggested that several common ADs confer an excess risk of LADA. The biological mechanisms involve innate and adaptive immune responses. Having concomitant ADs does not seem to affect the risk of retinopathy in LADA, but the poorer HbA_1c_ trajectories in LADA with AD suggest that there may be adverse long-term vascular consequences. Clinicians should be aware of the potential for LADA in individuals with ADs, suggesting the need for screening for GADA among individuals with newly diagnosed diabetes.

## Supplementary Information

Below is the link to the electronic supplementary material.

ESM (PDF 6222 KB)

## Data Availability

The datasets used in the current study are available from the corresponding author upon reasonable request. The functional pathway analysis used publicly available GWAS data.

## Abbreviations

AD: Autoimmune disease
CDR: Cause-of Death Register
GWAS: Genome-wide association studies
LADA: Latent autoimmune diabetes in adults
NDR: National Diabetes Register
NPR: National Patient Register
