# Supplementary material for: Autoimmune diseases and the risk and prognosis of latent autoimmune diabetes in adults
Source: Diabetologia. 2024 Oct 28;68(2):331–41. doi: 10.1007/s00125-024-06303-4 (PMC11732938; doi:10.1007/s00125-024-06303-4)
Supplement: Supplementary file 1 — ESM (PDF 6222 KB) [file 125_2024_6303_MOESM1_ESM.pdf]

Electronic supplementary materials (ESM)

List of ESM Tables

ESM Table 1. ICD codes for detecting autoimmune diseases in Swedish Patient Register and the Regional Primary Care Register of Scania 3
ESM Table 2. Data sources and ICD codes for diabetic retinopathy 5
ESM Table 3. Frequency of autoimmune disease of individuals with LADA and type 2 diabetes and controls 6
ESM Table 4. Autoimmune disease and risk of LADA and type 2 diabetes 8
ESM Table 5. Multiple testing correction for individual ADs and risk of LADA, LADA ^{high}, LADA ^{low}, and type 2 diabetes..........................10
ESM Table 6. Autoimmune disease and risk of LADA, LADA ^{high}, LADA ^{low}, and type 2 diabetes (drawn only from register data; thyroid dysfunction separated into Graves' and Hashimoto, inflammatory bowel disease into Crohn's disease and ulcerative colitis)..........................11
ESM Table 7. Autoimmune disease and risk of LADA ^{low} and LADA ^{high} 13
ESM Table 8. Autoimmune disease and risk of LADA and type 2 diabetes (drawn only from register data) 15
ESM Table 9. Autoimmune disease and risk of LADA ^{low} and LADA ^{high} (drawn only from register data) 17
ESM Table 10. Sensitivity analyses of autoimmune diseases without psoriasis and the risk of type 2 diabetes..........................19
ESM Table 11. Autoimmune disease in first-degree relatives and the risk of LADA and type 2 diabetes..........................20
ESM Table 12. Autoimmune disease in first-degree relatives and the risk of LADA ^{low} and LADA ^{high} 22
ESM Table 13. Multiple testing correction for ADs in first-degree relatives and risk of LADA, LADA ^{high}, LADA ^{low}, and type 2 diabetes..........................24
ESM Table 14. Interaction between having any autoimmune disease (AD) and first-degree relatives with AD on the risk of LADA and type 2 diabetes 25
ESM Table 15. Characteristics of individuals with LADA and type 2 diabetes by autoimmune disease status at baseline 26
ESM Table 16. Sensitivity analyses of incidence of diabetic retinopathy in people with LADA with and without autoimmune co-morbidity, compared to type 2 diabetes, additionally adjusting for glucose-lowering drugs, statin, and antihypertensives 27

List of ESM Figures

ESM Fig. 1. Study design and register linkage 28
ESM Fig. 2. Flowchart of the biological pathway analyses 29
ESM Fig. 3. Autoimmune disease and risk of LADA in men and women 30
ESM Fig. 4. Biological pathways between Crohn's disease and LADA 31
ESM Fig. 5. Biological pathways between ulcerative colitis and LADA 32
ESM Fig. 6. Biological pathways between Graves' disease and LADA 33
ESM Fig. 7. Biological pathways between hypothyroidism and LADA 34
ESM Fig. 8. Biological pathways between vitiligo and LADA 35

ESM Fig. 9. Biological pathways between hypothyroidism and LADA (excluding major histocompatibility complex) 36
ESM Fig. 10. Biological pathways between vitiligo and LADA (excluding major histocompatibility complex) 37
ESM Fig. 11. Glycated haemoglobin (HbA1c) trajectories for LADA with and without autoimmune diseases 38

ESM Table 1. ICD codes for detecting autoimmune diseases in Swedish Patient Register (ICD-9 and ICD-10) and the Regional Primary Care Register of Scania (ICD-10-P)

	**ICD-9**	**ICD-10**	**ICD-10-P**
Addison's disease	255E	E27.1, E27.2	E271
Amyotrophic lateral sclerosis	335C	G12.2	G122
Ankylosing spondylitis	720A	M45, M08.1	M45-
Autoimmune hemolytic anemia	283A	D59.0	
Behcet disease	136B	M35.2	
Coeliac disease	579A	K90.0	K900
Chorea minor	392X	I02.9	
Chronic rheumatic heart disease	393-398	I05-I09	
Crohn's disease	555	K50	K50-
Discoid lupus erythematosus	695E	L93.0	
Graves'/hyperthyroidism	242	E05	E05-
Hashimoto/hypothroidism	245C, 243, 244	E00-E03, E06.3	E01-P, E03-
Immune thrombocytopenic purpura	287D	D69.3	
Localized scleroderma	701A	L94.0	
Lupoid hepatitis	571EJ	K75.4	
Multiple sclerosis	340	G35	G35-
Myasthenia gravis	358A	G70.0	G700
Pernicious anemia	281A	D51.0	
Polyarteritis nodosa	446A	M30.0	
Polymyalgia rheumatica	725	M315, M35.3	M353
Polymyositis/dermatomyositis	710D, 710E	M33	
Primary biliary cirrhosis	571G	K74.3	
Psoriasis	696	L40	L409P, L403
Reiter disease	099D, 711B	M02.3	
Rheumatic fever	390-392, excluded 392X	I00-02, excluded I02.9	
Rheumatoid arthritis	714, excluded 714E, 714X	M05, M06, M08.0, M08.2	
Sarcoidosis	135	D86	D86-

Sjögren's syndrome	710C	M35.0	M350
Systemic lupus erythematosus	710A	M32	
Systemic sclerosis	710B	M34	
Ulcerative colitis	556	K51	K51-
Wegener's granulomatosis	446E	M31.3	
Vitiligo		L80	L80-

ESM Table 2. Data sources and ICD codes for diabetic retinopathy

Data source	Diabetic retinopathy	ICD-10
National Diabetes Register	Severe non-proliferative retinopathy	
	Pre-proliferative diabetic retinopathy	
	Proliferative diabetic retinopathy	
National Patient Register	Pre-proliferative diabetic retinopathy	E10.3B, E11.3B, E12.3B, E14.3B
	Proliferative diabetic retinopathy	H36.0B, E10.3C, E11.3C, E12.3C, E13.3C, E14.3C
	Diabetes with advanced eye disease	E10.3D-W, E11.3D-W, E12.3D-W, E14.3D-W
	Other proliferative retinopathy	H35.2
	Diabetic cataract	H28.0
	Retinal haemorrhage	H35.6
	Visual impairment	H54
	Vitreous haemorrhage	H43.1
Cause of Death Register	Death from diabetic retinopathy	E10.3, E11.3, E12.3, E13.3, E14.3, H28.0, H36.0

ESM Table 3. Frequency of autoimmune disease of individuals with LADA and type 2 diabetes and controls

**Type of autoimmune disease, n (%)**	**Control (N = 2355)**	**LADA (N = 586)**	**Type 2 diabetes (N = 2003)**	***p***	**LADA ^{low} (N=277)**	**LADA ^{high} (N=298)**	***p***
Coeliac disease	37 (1.6)	6 (1.0)	22 (1.1)	0.878	2 (0.7)	4 (1.3)	0.687
Inflammatory bowel disease	48 (2.0)	21 (3.6)	43 (2.1)	0.049	8 (2.9)	13 (4.4)	0.346
Multiple sclerosis	9 (0.4)	4 (0.7)	11 (0.5)	0.757	3 (1.1)	1 (0.3)	0.356
Psoriasis	133 (5.6)	44 (7.5)	172 (8.6)	0.406	21 (7.6)	21 (7.0)	0.806
Rheumatoid arthritis	90 (3.8)	38 (6.5)	90 (4.5)	0.050	20 (7.2)	17 (5.7)	0.459
Sjögren's syndrome	21 (0.9)	7 (1.2)	15 (0.7)	0.308	4 (1.4)	3 (1.0)	0.716
Systemic lupus erythematosus	7 (0.3)	1 (0.2)	8 (0.4)	0.694	1 (0.4)	0 (0.0)	0.482
Thyroid dysfunction	203 (8.6)	104 (17.7)	216 (10.8)	<0.001	33 (11.9)	66 (22.1)	0.001
Vitiligo	21 (0.9)	19 (3.2)	19 (0.9)	<0.001	4 (1.4)	15 (5.0)	0.016
Addison's disease	0 (0.0)	2 (0.3)	1 (0.0)	0.130	0 (0.0)	2 (0.7)	0.500
Amyotrophic lateral sclerosis	2 (0.1)	0 (0.0)	0 (0.0)	NA	0 (0.0)	0 (0.0)	NA
Ankylosing spondylitis	10 (0.4)	1 (0.2)	14 (0.7)	0.215	0 (0.0)	1 (0.3)	1.000
Autoimmune hemolytic anemia	0 (0.0)	0 (0.0)	0 (0.0)	NA	0 (0.0)	0 (0.0)	NA
Behcet disease	0 (0.0)	0 (0.0)	0 (0.0)	NA	0 (0.0)	0 (0.0)	NA
Chorea minor	0 (0.0)	0 (0.0)	0 (0.0)	NA	0 (0.0)	0 (0.0)	NA
Chronic rheumatic heart disease	0 (0.0)	1 (0.2)	3 (0.1)	1.000	0 (0.0)	1 (0.3)	1.000
Discoid lupus erythematosus	3 (0.1)	1 (0.2)	0 (0.0)	0.226	1 (0.4)	0 (0.0)	0.482
Immune thrombocytopenic purpura	0 (0.0)	0 (0.0)	0 (0.0)	NA	0 (0.0)	0 (0.0)	NA
Localized scleroderma	1 (0.0)	0 (0.0)	1 (0.0)	1.000	0 (0.0)	0 (0.0)	NA
Lupoid hepatitis	0 (0.0)	0 (0.0)	2 (0.1)	1.000	0 (0.0)	0 (0.0)	NA
Myasthenia gravis	1 (0.0)	0 (0.0)	3 (0.1)	1.000	0 (0.0)	0 (0.0)	NA
Pernicious anemia	1 (0.0)	1 (0.2)	0 (0.0)	0.226	0 (0.0)	1 (0.3)	1.000
Polyarteritis nodosa	0 (0.0)	0 (0.0)	0 (0.0)	NA	0 (0.0)	0 (0.0)	NA
Polymyalgia rheumatica	28 (1.2)	10 (1.7)	39 (1.9)	0.707	2 (0.7)	8 (2.7)	0.109
Polymyositis/dermatomyositis	1 (0.0)	1 (0.2)	0 (0.0)	0.226	1 (0.4)	0 (0.0)	0.482
Primary biliary cirrhosis	2 (0.1)	1 (0.2)	2 (0.1)	0.537	1 (0.4)	0 (0.0)	0.482
Reiter disease	0 (0.0)	0 (0.0)	1 (0.0)	1.000	0 (0.0)	0 (0.0)	NA

Rheumatic fever	1 (0.0)	0 (0.0)	2 (0.1)	1.000	0 (0.0)	0 (0.0)	NA
Sarcoidosis	7 (0.3)	2 (0.3)	11 (0.5)	0.745	2 (0.7)	0 (0.0)	0.232
Systemic sclerosis	0 (0.0)	1 (0.2)	3 (0.1)	1.000	0 (0.0)	1 (0.3)	1.000
Wegener's granulomatosis	0 (0.0)	1 (0.2)	0 (0.0)	0.226	1 (0.4)	0 (0.0)	0.482

LADA = latent autoimmune diabetes in adults.

LADA ^{low} was defined as those with glutamic acid decarboxylase antibodies (GADA) <250 U/ml, while LADA ^{high} was those with GADA ≥250 U/ml.
The P-values are for LADA vs. type 2 diabetes, and LADA ^{high} vs. LADA ^{low}.

ESM Table 4. Autoimmune disease and risk of LADA and type 2 diabetes. Odds ratios (ORs) and 95% confidence intervals (CIs).

	**LADA**				**Type 2 diabetes**			
	No cases/ controls	Model 1 OR (95% CI)	Model 2 OR (95% CI)	Model 3 OR (95% CI)	No cases/ controls	Model 1 OR (95% CI)	Model 2 OR (95% CI)	Model 3 OR (95% CI)
Any AD								
No	409/1861	Ref	Ref	Ref	1524/1861	Ref	Ref	Ref
Yes	177/494	1.79 (1.45-2.21)	1.82 (1.46-2.26)	1.70 (1.36-2.13)	479/494	1.26 (1.08-1.47)	1.14 (0.95-1.36)	1.18 (0.98-1.42)
Number of ADs								
0	409/1861	Ref	Ref	Ref	1524/1861	Ref	Ref	Ref
1	131/402	1.63 (1.30-2.06)	1.69 (1.33-2.14)	1.59 (1.25-2.03)	375/402	1.21 (1.03-1.43)	1.10 (0.91-1.34)	1.14 (0.93-1.39)
2+	46/92	2.53 (1.73-3.70)	2.37 (1.60-3.51)	2.19 (1.46-3.28)	104/92	1.49 (1.10-2.01)	1.30 (0.91-1.84)	1.38 (0.96-1.98)
continuous		*1.55 (1.34-1.79)*	*1.53 (1.32-1.78)*	*1.47 (1.26-1.71)*		*1.22 (1.09-1.36)*	*1.13 (1.00-1.29)*	*1.17 (1.03-1.34)*
**Type of AD**								
Coeliac disease								
No	582/2321	Ref	Ref	Ref	1986/2321	Ref	Ref	Ref
Yes	4/34	0.43 (0.15-1.25)	0.52 (0.18-1.54)	0.54 (0.18-1.59)	17/34	0.63 (0.35-1.16)	0.76 (0.38-1.56)	0.81 (0.40-1.65)
Inflammatory bowel disease								
No	566/2309	Ref	Ref	Ref	1961/2309	Ref	Ref	Ref
Yes	20/46	1.75 (1.01-3.03)	1.86 (1.06-3.26)	1.78 (1.00-3.16)	42/26	1.22 (0.79-1.90)	1.32 (0.79-2.20)	1.39 (0.83-2.35)
Polymyalgia rheumatica								
No	578/2329	Ref	Ref	Ref	1977/2329	Ref	Ref	Ref
Yes	8/26	1.06 (0.46-2.48)	0.97 (0.40-2.35)	0.80 (0.33-1.94)	26/26	0.80 (0.45-1.43)	0.84 (0.44-1.59)	0.73 (0.38-1.40)
Psoriasis								
No	543/2226	Ref	Ref	Ref	1839/2226	Ref	Ref	Ref
Yes	43/129	1.35 (0.93-1.95)	1.22 (0.83-1.79)	1.22 (0.83-1.80)	164/129	1.54 (1.20-1.98)	1.33 (0.99-1.78)	1.47 (1.08-1.99)
Rheumatoid arthritis								
No	549/2267	Ref	Ref	Ref	1917/2267	Ref	Ref	Ref
Yes	37/88	1.66 (1.09-2.53)	1.51 (0.97-2.34)	1.47 (0.94-2.30)	86/88	1.02 (0.74-1.40)	0.84 (0.59-1.22)	0.85 (0.58-1.25)
Sjögren's syndrome								
No	579/2335	Ref	Ref	Ref	1989/2335	Ref	Ref	Ref
Yes	7/20	0.95 (0.36-2.45)	0.90 (0.33-2.43)	0.80 (0.28-2.25)	14/20	0.77 (0.37-1.60)	0.64 (0.28-1.48)	0.63 (0.26-1.49)
Thyroid dysfunction								
No	501/2159	Ref	Ref	Ref	1809/2159	Ref	Ref	Ref
Yes	85/196	2.01 (1.51-2.69)	2.09 (1.55-2.82)	1.88 (1.38-2.56)	194/196	1.39 (1.11-1.74)	1.31 (1.01-1.71)	1.30 (0.99-1.70)
Vitiligo								

No	567/2337	Ref	Ref	Ref	1985/2337	Ref	Ref	Ref
Yes	19/18	3.68 (1.89-7.17)	3.78 (1.90-7.53)	3.91 (1.93-7.94)	18/18	1.01 (0.51-2.00)	1.01 (0.45-2.24)	1.10 (0.48-2.50)
Any other ADs								
No	573/2314	Ref	Ref	Ref	1951/2314	Ref	Ref	Ref
Yes	13/41	1.10 (0.56-2.14)	1.14 (0.58-2.25)	1.34 (0.67-2.66)	52/41	1.43 (0.93-2.20)	1.47 (0.90-2.43)	1.66 (0.99-2.81)

AD = autoimmune disease. LADA = latent autoimmune disease in adults.

Model 1: adjusted for age and sex. Model 2: adjusted for age, sex, education, smoking, physical activity, and body mass index (BMI). Model 3: adjusted for age, sex, education, smoking, physical activity, BMI, family history of type 1 diabetes, family history of type 2 diabetes, and family history of any AD. ORs for individual ADs were mutually adjusted for the other ADs.

ESM Table 5. Multiple testing correction for the associations between individual ADs and risk of LADA, LADA ^{high}, LADA ^{low}, and type 2 diabetes

	**LADA**		**LADA ^{high}**		**LADA ^{low}**		**Type 2 diabetes**	
	Original P-value	Adjusted P-value	Original P-value	Adjusted P-value	Original P-value	Adjusted P-value	Original P-value	Adjusted P-value
Coeliac disease	0.2653150	1	0.858073	1	-	-	0.5605900	1
Inflammatory bowel disease	0.0495500	0.4459500	0.030848	0.277632	0.3285100	1	0.2119500	1
Polymyalgia rheumatica	0.6222200	1	0.305335	1	0.0990500	0.7924000	0.3403600	1
Psoriasis	0.3114060	1	0.576106	1	0.2570400	1	0.0131400	0.1182600
Rheumatoid arthritis	0.0942450	0.8482050	0.595168	1	0.0208900	0.1671200	0.4124500	1
Sjögren's syndrome	0.6648760	1	0.429866	1	0.5521400	1	0.2917000	1
Thyroid dysfunction	0.0000586	0.0005274	0.000001	0.000012	0.4859800	1	0.0547000	0.4923000
Vitiligo	0.0001570	0.0014130	0.000018	0.000160	0.1506700	1	0.8273800	1
Any other ADs	0.4097230	1	0.612152	1	0.1003300	0.8026400	0.0562900	0.5066100

AD = autoimmune disease. LADA = latent autoimmune diabetes in adults.

LADA ^{low} was defined as those with glutamic acid decarboxylase antibodies (GADA) <250 U/ml, while LADA ^{high} was those with GADA ≥250 U/ml.

The original P-values were derived from models adjusted for age, sex, education, smoking, physical activity, body mass index, family history of type 1 diabetes, family history of type 2 diabetes, family history of any AD, and individual ADs mutually adjusted for each other. Adjusted P-values were obtained by multiplying the original P-values by nine for the outcomes of LADA, LADA ^{high}, and type 2 diabetes, and by eight for the outcome of LADA ^{low}.

ESM Table 6. Autoimmune disease and risk of LADA, LADA ^{high}, LADA ^{low}, and type 2 diabetes (drawn only from register data; thyroid dysfunction separated into Graves' and Hashimoto, inflammatory bowel disease into Crohn's disease and ulcerative colitis). Odds ratios (ORs) and 95% confidence intervals (CIs).

	**LADA**	**LADA ^{high}**	**LADA ^{low}**	**Type 2 diabetes**
	OR (95% CI)	OR (95% CI)	OR (95% CI)	OR (95% CI)
**Type of AD**				
Coeliac disease				
No	Ref	Ref		Ref
Yes	0.81 (0.17-3.76)	1.41 (0.30-6.66)	-	0.92 (0.28-3.08)
Crohn's disease				
No	Ref	Ref	Ref	Ref
Yes	1.57 (0.56-4.40)	1.26 (0.32-4.96)	2.25 (0.60-8.47)	1.98 (0.77-5.07)
Graves'				
No	Ref	Ref	Ref	Ref
Yes	2.35 (1.12-4.95)	1.91 (0.77-4.74)	2.26 (0.72-7.15)	1.73 (0.79-3.78)
Hashimoto				
No	Ref		Ref	Ref
Yes	1.66 (1.12-2.47)	2.10 (1.32-3.34)	1.20 (0.65-2.22)	1.11 (0.78-1.56)
Polymyalgia rheumatica				
No	Ref		Ref	Ref
Yes	0.80 (0.33-1.95)	1.43 (0.55-3.72)	0.21 (0.03-1.61)	0.76 (0.40-1.44)
Psoriasis				
No	Ref		Ref	Ref
Yes	1.38 (0.86-2.20)	1.19 (0.63-2.24)	1.74 (0.96-3.18)	1.45 (0.99-2.11)
Rheumatoid arthritis				
No	Ref		Ref	Ref
Yes	1.56 (0.77-3.16)	1.67 (0.69-4.06)	1.42 (0.54-3.71)	0.82 (0.42-1.60)
Sjögren's syndrome				
No	Ref		Ref	Ref
Yes	0.45 (0.10-2.11)	-	1.21 (0.24-6.07)	0.60 (0.20-1.77)
Ulcerative colitis				
No	Ref	Ref		Ref
Yes	0.93 (0.36-2.43)	1.95 (0.74-5.12)	-	1.18 (0.57-2.45)
Vitiligo				
No	Ref	Ref	Ref	Ref
Yes	8.55 (2.20-33.18)	13.17 (3.27-53.05)	3.29 (0.33-32.91)	1.77 (0.35-8.94)
Any other ADs				

No	Ref	Ref	Ref	Ref
Yes	1.50 (0.76-3.00)	0.82 (0.28-2.36)	2.31 (1.01-5.32)	1.43 (0.82-2.48)

AD = autoimmune disease. LADA = latent autoimmune disease in adults.
LADA ^{low} was defined as those with glutamic acid decarboxylase antibodies (GADA) <250 U/ml, while LADA ^{high} was those with GADA ≥250 U/ml.
Models were adjusted for age, sex, education, smoking, physical activity, BMI, family history of type 1 diabetes, family history of type 2 diabetes, family history of any AD, and individual ADs mutually adjusted for each other. For the investigation of individual AD and the risk of LADA ^{low}, coeliac disease and ulcerative colitis were excluded as there were no cases. For the investigation of individual AD and the risk of LADA ^{high}, Sjögren's syndrome was excluded as there were no cases.

ESM Table 7. Autoimmune disease and risk of LADA low and LADA high. Odds ratios (ORs) and 95% confidence intervals (CIs).

	LADA low				LADA high			
		Model 1	Model 2	Model 3		Model 1	Model 2	Model 3
	No cases/ controls	OR (95% CI)	OR (95% CI)	OR (95% CI)	No cases/ controls	OR (95% CI)	OR (95% CI)	OR (95% CI)
Any AD								
No	207/1861	Ref	Ref	Ref	195/1861	Ref	Ref	Ref
Yes	70/494	1.49 (1.10-2.02)	1.56 (1.14-2.13)	1.55 (1.12-2.13)	103/494	2.09 (1.59-2.74)	2.10 (1.60-2.77)	1.93 (1.45-2.57)
Number of ADs								
0	207/1861	Ref	Ref	Ref	195/1861	Ref	Ref	Ref
1	53/402	1.38 (0.99-1.92)	1.48 (1.05-2.08)	1.48 (1.04-2.09)	75/402	1.88 (1.40-2.54)	1.90 (1.40-2.58)	1.75 (1.28-2.39)
2 or more	17/92	2.08 (1.19-3.63)	1.90 (1.07-3.38)	1.83 (1.02-3.31)	28/92	3.05 (1.91-4.86)	3.01 (1.87-4.84)	2.75 (1.69-4.48)
continuous		*1.38 (1.12-1.70)*	*1.37 (1.11-1.69)*	*1.36 (1.09-1.69)*		*1.70 (1.42-2.03)*	*1.68 (1.40-2.02)*	*1.61 (1.33-1.94)*
**Type of AD**								
Coeliac disease								
No	277/2321				294/2321	Ref	Ref	Ref
Yes	0/34	-	-	-	4/34	0.75 (0.25-2.23)	0.86 (0.29-2.62)	0.90 (0.30-2.72)
Inflammatory bowel disease								
No	270/2309	Ref	Ref	Ref	285/2309	Ref	Ref	Ref
Yes	7/46	1.42 (0.63-3.24)	1.54 (0.66-3.56)	1.53 (0.65-3.57)	13/46	2.08 (1.07-4.02)	2.24 (1.14-4.37)	2.13 (1.07-4.24)
Polymyalgia rheumatica								
No	276/2329	Ref	Ref	Ref	291/2329	Ref	Ref	Ref
Yes	1/26	0.26 (0.03-1.99)	0.19 (0.02-1.62)	0.17 (0.02-1.39)	7/26	2.13 (0.86-5.26)	1.98 (0.77-5.07)	1.64 (0.64-4.20)
Psoriasis								
No	256/2226	Ref	Ref	Ref	278/2226	Ref	Ref	Ref
Yes	21/129	1.34 (0.82-2.20)	1.25 (0.75-2.07)	1.35 (0.81-2.25)	20/129	1.29 (0.78-2.13)	1.20 (0.72-2.01)	1.16 (0.69-1.96)
Rheumatoid arthritis								
No	258/2267	Ref	Ref	Ref	281/2267	Ref	Ref	Ref
Yes	19/88	2.05 (1.18-3.54)	1.94 (1.09-3.43)	1.98 (1.11-3.55)	17/88	1.40 (0.78-2.52)	1.26 (0.69-2.30)	1.18 (0.64-2.17)
Sjögren's syndrome								
No	273/2335	Ref	Ref	Ref	295/2335	Ref	Ref	Ref
Yes	4/20	1.74 (0.55-5.50)	1.61 (0.49-5.24)	1.45 (0.43-4.91)	3/20	0.57 (0.14-2.34)	0.49 (0.11-2.18)	0.54 (0.12-2.51)
Thyroid dysfunction								
No	252/2159	Ref	Ref	Ref	241/2159	Ref	Ref	Ref
Yes	25/196	1.26 (0.80-2.00)	1.34 (0.83-2.17)	1.19 (0.73-1.95)	57/196	2.64 (1.86-3.75)	2.73 (1.91-3.91)	2.46 (1.71-3.55)
Vitiligo								

No	273/2337	Ref	Ref	Ref	283/2337	Ref	Ref	Ref
Yes	4/18	1.70 (0.56-5.14)	1.92 (0.62-5.97)	2.31 (0.74-7.27)	15/18	5.94 (2.86-12.35)	5.82 (2.74-12.39)	5.46 (2.51-11.84)
Any other ADs								
No	269/2314	Ref	Ref	Ref	293/2314	Ref	Ref	Ref
Yes	8/41	1.66 (0.74-3.71)	1.73 (0.76-3.94)	2.01 (0.87-4.60)	5/41	0.65 (0.23-1.81)	0.67 (0.24-1.88)	0.76 (0.26-2.19)

AD = autoimmune disease. LADA = latent autoimmune disease in adults.

LADA ^{low} was defined as those with glutamic acid decarboxylase antibodies (GADA) <250 U/ml, while LADA ^{high} was those with GADA ≥250 U/ml.

Model 1: adjusted for age and sex. Model 2: adjusted for age, sex, education, smoking, physical activity, and body mass index (BMI). Model 3: adjusted for age, sex, education, smoking, physical activity, BMI, family history of type 1 diabetes, family history of type 2 diabetes, and family history of any AD. ORs for individual ADs were mutually adjusted for the other ADs. For the investigation of individual AD and the risk of LADA ^{low}, coeliac disease was excluded as there were no cases.

ESM Table 8. Autoimmune disease and risk of LADA and type 2 diabetes (drawn only from register data). Odds ratios (ORs) and 95% confidence intervals (CIs).

	LADA				Type 2 diabetes			
		Model 1	Model 2	Model 3		Model 1	Model 2	Model 3
	No cases/ controls	OR (95% CI)	OR (95% CI)	OR (95% CI)	No cases/ controls	OR (95% CI)	OR (95% CI)	OR (95% CI)
Any AD								
No	461/2055	Ref	Ref	Ref	1675/2055	Ref	Ref	Ref
Yes	125/300	2.00 (1.57-2.54)	1.98 (1.54-2.53)	1.81 (1.41-2.33)	328/300	1.38 (1.16-1.65)	1.18 (0.96-1.45)	1.20 (0.97-1.49)
Number of ADs								
0	461/2055	Ref	Ref	Ref	1675/2055	Ref	Ref	Ref
1	98/238	1.97 (1.52-2.57)	1.99 (1.52-2.61)	1.82 (1.38-2.40)	271/238	1.44 (1.19-1.75)	1.20 (0.96-1.51)	1.22 (0.96-1.54)
2+	27/62	2.09 (1.31-3.34)	1.92 (1.18-3.11)	1.77 (1.08-2.91)	57/62	1.16 (0.80-1.70)	1.09 (0.71-1.69)	1.14 (0.73-1.78)
continuous		*1.60 (1.35-1.91)*	*1.57 (1.31-1.88)*	*1.49 (1.24-1.79)*		*1.25 (1.09-1.42)*	*1.15 (0.99-1.33)*	*1.18 (1.01-1.38)*
**Type of AD**								
Coeliac disease								
No	584/2341	Ref	Ref	Ref	1997/2341	Ref	Ref	Ref
Yes	2/14	0.55 (0.12-2.47)	0.84 (0.19-3.79)	0.84 (0.18-3.95)	6/14	0.47 (0.18-1.25)	0.84 (0.25-2.78)	0.97 (0.29-3.21)
Inflammatory bowel disease								
No	573/2319	Ref	Ref	Ref	1973/2319	Ref	Ref	Ref
Yes	13/36	1.34 (0.68-2.61)	1.36 (0.69-2.70)	1.26 (0.62-2.53)	30/36	1.07 (0.64-1.78)	1.22 (0.68-2.21)	1.29 (0.71-2.36)
Polymyalgia rheumatica								
No	578/2329	Ref	Ref	Ref	1977/2329	Ref	Ref	Ref
Yes	8/26	1.02 (0.43-2.39)	0.93 (0.38-2.27)	0.77 (0.32-1.88)	26/26	0.82 (0.46-1.45)	0.85 (0.45-1.59)	0.73 (0.39-1.40)
Psoriasis								
No	555/2285	Ref	Ref	Ref	1894/2285	Ref	Ref	Ref
Yes	31/70	1.66 (1.06-2.59)	1.41 (0.89-2.25)	1.38 (0.86-2.20)	109/70	1.82 (1.33-2.50)	1.41 (0.97-2.03)	1.47 (1.00-2.14)
Rheumatoid arthritis								
No	573/2325	Ref	Ref	Ref	1979/2325	Ref	Ref	Ref
Yes	13/30	1.76 (0.89-3.49)	1.81 (0.89-3.66)	1.55 (0.77-3.14)	24/30	0.79 (0.45-1.40)	0.79 (0.41-1.51)	0.82 (0.42-1.60)
Sjögren's syndrome								
No	584/2341	Ref	Ref	Ref	1995/2341	Ref	Ref	Ref
Yes	2/14	0.46 (0.10-2.16)	0.40 (0.08-1.92)	0.44 (0.09-2.07)	8/14	0.79 (0.31-1.98)	0.56 (0.19-1.60)	0.59 (0.20-1.75)
Thyroid dysfunction								
No	525/2236	Ref	Ref	Ref	1870/2236	Ref	Ref	Ref
Yes	61/119	2.34 (1.67-3.28)	2.34 (1.66-3.32)	2.16 (1.51-3.08)	133/119	1.51 (1.15-1.98)	1.26 (0.91-1.73)	1.26 (0.91-1.74)

Vitiligo								
No	577/2352	Ref	Ref	Ref	1999/2352	Ref	Ref	Ref
Yes	9/3	9.39 (2.49-35.37)	9.94 (2.60-37.99)	8.92 (2.31-34.45)	4/3	1.37 (0.30-6.21)	2.03 (0.40-10.24)	1.76 (0.35-8.91)
Any other ADs								
No	573/2317	Ref	Ref	Ref	1958/2317	Ref	Ref	Ref
Yes	13/38	1.23 (0.63-2.43)	1.23 (0.62-2.45)	1.47 (0.73-2.93)	45/38	1.33 (0.84-2.09)	1.24 (0.73-2.09)	1.42 (0.82-2.46)

AD = autoimmune disease. LADA = latent autoimmune disease in adults.

Model 1: adjusted for age and sex. Model 2: adjusted for age, sex, education, smoking, physical activity, and body mass index (BMI). Model 3: adjusted for age, sex, education, smoking, physical activity, BMI, family history of type 1 diabetes, family history of type 2 diabetes, and family history of any AD. ORs for individual ADs were mutually adjusted for the other ADs.

ESM Table 9. Autoimmune disease and risk of LADA low and LADA high (drawn only from register data). Odds ratios (ORs) and 95% confidence intervals (CIs).

	**LADA low**				**LADA high**			
		Model 1	Model 2	Model 3		Model 1	Model 2	Model 3
	No cases/ controls	OR (95% CI)	OR (95% CI)	OR (95% CI)	No cases/ controls	OR (95% CI)	OR (95% CI)	OR (95% CI)
Any AD								
No	229/2055	Ref	Ref	Ref	224/2055	Ref	Ref	Ref
Yes	48/300	1.66 (1.17-2.34)	1.66 (1.16-2.37)	1.56 (1.09-2.25)	74/300	2.32 (1.72-3.14)	2.32 (1.71-3.15)	2.13 (1.56-2.91)
Number of ADs								
0	229/2055	Ref	Ref	Ref	224/2055	Ref	Ref	Ref
1	38/238	1.63 (1.12-2.39)	1.69 (1.14-2.50)	1.59 (1.07-2.36)	58/238	2.31 (1.67-3.21)	2.35 (1.68-3.27)	2.15 (1.53-3.02)
2+	10/62	1.75 (0.87-3.52)	1.53 (0.74-3.16)	1.48 (0.71-3.08)	16/62	2.36 (1.33-4.20)	2.24 (1.24-4.03)	2.07 (1.14-3.76)
continuous		*1.43 (1.12-1.84)*	*1.40 (1.08-1.81)*	*1.36 (1.05-1.77)*		*1.73 (1.40-2.14)*	*1.71 (1.38-2.12)*	*1.63 (1.31-2.04)*
**Type of AD**								
Coeliac disease								
No	277/2341				296/2341	Ref	Ref	Ref
Yes	0/14	-	-	-	2/14	0.95 (0.20-4.41)	1.31 (0.28-6.14)	1.40 (0.30-6.64)
Inflammatory bowel disease								
No	274/2319	Ref	Ref	Ref	288/2319	Ref	Ref	Ref
Yes	3/36	0.83 (0.25-2.75)	0.85 (0.25-2.87)	0.81 (0.24-2.76)	10/36	1.84 (0.86-3.98)	1.99 (0.92-4.31)	1.82 (0.82-4.04)
Polymyalgia rheumatica								
No	276/2329	Ref	Ref	Ref	291/2329			
Yes	1/26	0.28 (0.04-2.15)	0.24 (0.03-1.84)	0.20 (0.03-1.56)	7/26	1.84 (0.73-4.62)	1.68 (0.64-4.37)	1.39 (0.53-3.63)
Psoriasis								
No	261/2285	Ref	Ref	Ref	284/2285			
Yes	16/70	1.89 (1.06-3.35)	1.64 (0.90-2.99)	1.73 (0.95-3.16)	14/70	1.46 (0.79-2.69)	1.29 (0.69-2.44)	1.18 (0.62-2.23)
Rheumatoid arthritis								
No	271/2325	Ref	Ref	Ref	291/2325			
Yes	6/30	1.49 (0.59-3.81)	1.51 (0.57-3.98)	1.38 (0.53-3.61)	7/30	1.97 (0.83-4.69)	1.91 (0.78-4.66)	1.68 (0.69-4.08)
Sjögren's syndrome								
No	275/2341	Ref	Ref	Ref	298/2341			
Yes	2/14	1.25 (0.26-5.98)	1.13 (0.23-5.56)	1.25 (0.26-6.07)	0/14	-	-	-
Thyroid dysfunction								
No	258/2236	Ref	Ref	Ref	259/2236	Ref	Ref	Ref
Yes	19/119	1.65 (0.97-2.78)	1.64 (0.95-2.83)	1.43 (0.82-2.51)	39/119	2.73 (1.82-4.10)	2.79 (1.84-4.21)	2.66 (1.75-4.04)
Vitiligo								

No	276/2352	Ref	Ref	Ref	290/2352	Ref	Ref	Ref
Yes	1/3	2.45 (0.25-24.11)	3.00 (0.30-30.27)	3.28 (0.33-32.75)	8/3	13.98 (3.57-54.67)	14.22 (3.59-56.34)	12.22 (3.06-48.82)
Any other ADs								
No	269/2317	Ref	Ref	Ref	293/2317	Ref	Ref	Ref
Yes	8/38	1.94 (0.87-4.36)	1.98 (0.87-4.50)	2.30 (1.01-5.26)	5/38	0.66 (0.23-1.86)	0.66 (0.23-1.91)	0.79 (0.27-2.30)

AD = autoimmune disease. LADA = latent autoimmune disease in adults.

LADA ^{low} was defined as those with glutamic acid decarboxylase antibodies (GADA) <250 U/ml, while LADA ^{high} was those with GADA ≥250 U/ml.

Model 1: adjusted for age and sex. Model 2: adjusted for age, sex, education, smoking, physical activity, and body mass index (BMI). Model 3: adjusted for age, sex, education, smoking, physical activity, BMI, family history of type 1 diabetes, family history of type 2 diabetes, and family history of any AD. ORs for individual ADs were mutually adjusted for the other ADs. For the investigation of individual AD and the risk of LADA ^{low}, coeliac disease was excluded as there were no cases. For the investigation of individual AD and the risk of LADA ^{high}, Sjögren's syndrome was excluded as there were no cases.

ESM Table 10. Sensitivity analyses of autoimmune diseases without psoriasis and the risk of type 2 diabetes

	No cases/controls	Model 1<br>OR (95% CI)	Model 2<br>OR (95% CI)	Model 3<br>OR (95% CI)
Any AD (without psoriasis)				
No	1643/1955	Ref	Ref	Ref
Yes	360/400	1.15 (0.97-1.36)	1.07 (0.88-1.30)	1.08 (0.88-1.32)
Number of ADs (without psoriasis)				
0	1643/1955	Ref	Ref	Ref
1	287/338	1.09 (0.91-1.31)	1.01 (0.82-1.25)	1.02 (0.82-1.27)
2+	73/62	1.48 (1.03-2.12)	1.36 (0.90-2.05)	1.41 (0.92-2.16)
continuous	*NA*	*1.16 (1.02-1.32)*	*1.10 (0.95-1.27)*	*1.12 (0.96-1.30)*

AD = autoimmune disease.

Model 1: adjusted for age and sex. Model 2: adjusted for age, sex, education, smoking, physical activity, and body mass index (BMI). Model 3: adjusted for age, sex, education, smoking, physical activity, BMI, family history of type 1 diabetes, family history of type 2 diabetes, and family history of any AD.

ESM Table 11. Autoimmune disease in first-degree relatives and the risk of LADA and type 2 diabetes. Odds ratios (OR) and 95% confidence intervals (CI).

	LADA				Type 2 diabetes			
	No cases/ controls	Model 1 OR (95% CI)	Model 2 OR (95% CI)	Model 3 OR (95% CI)	No cases/ controls	Model 1 OR (95% CI)	Model 2 OR (95% CI)	Model 3 OR (95% CI)
Family history of any AD								
No	361/1574	Ref	Ref	Ref	1367/1574	Ref	Ref	Ref
Yes	225/781	1.17 (0.96-1.42)	1.17 (0.96-1.43)	1.08 (0.88-1.32)	636/781	0.98 (0.86-1.12)	0.98 (0.84-1.15)	0.90 (0.76-1.06)
Number of ADs in the family								
0	361/1574	Ref	Ref	Ref	1367/1574	Ref	Ref	Ref
1	156/618	1.03 (0.83-1.28)	1.04 (0.83-1.29)	0.96 (0.76-1.20)	503/618	0.97 (0.84-1.12)	0.97 (0.82-1.15)	0.88 (0.73-1.05)
2+	69/163	1.70 (1.24-2.33)	1.69 (1.22-2.34)	1.54 (1.10-2.14)	133/163	1.01 (0.79-1.30)	1.04 (0.78-1.39)	0.98 (0.73-1.33)
continuous		*1.19 (1.05-1.35)*	*1.19 (1.05-1.36)*	*1.14 (1.00-1.31)*		*0.99 (0.90-1.09)*	*1.00 (0.89-1.11)*	*0.95 (0.85-1.07)*
Number of relatives with any AD								
0	361/1574	Ref	Ref	Ref	1367/1574	Ref	Ref	Ref
1	168/649	1.07 (0.87-1.32)	1.07 (0.86-1.33)	0.98 (0.78-1.22)	526/649	0.97 (0.84-1.12)	0.98 (0.83-1.15)	0.89 (0.75-1.06)
2+	57/132	1.68 (1.19-2.37)	1.68 (1.17-2.39)	1.57 (1.09-2.26)	110/132	1.02 (0.77-1.34)	1.03 (0.74-1.42)	0.93 (0.66-1.31)
continuous		*1.20 (1.04-1.39)*	*1.20 (1.04-1.39)*	*1.14 (0.98-1.33)*		*1.00 (0.90-1.11)*	*1.01 (0.89-1.14)*	*0.94 (0.83-1.07)*
**Type of AD in the family**								
Coeliac disease								
No	571/2308	Ref	Ref	Ref	1965/2308	Ref	Ref	Ref
Yes	15/47	1.26 (0.69-2.32)	1.57 (0.84-2.93)	1.43 (0.75-2.73)	38/47	1.05 (0.67-1.64)	1.26 (0.73-2.17)	1.10 (0.63-1.93)
Inflammatory bowel disease								
No	577/2301	Ref	Ref	Ref	1972/2301	Ref	Ref	Ref
Yes	9/54	0.56 (0.27-1.16)	0.61 (0.29-1.29)	0.55 (0.26-1.18)	31/54	0.73 (0.46-1.16)	0.67 (0.39-1.17)	0.65 (0.37-1.14)
Multiple sclerosis								
No	575/2325	Ref	Ref	Ref	1982/2325	Ref	Ref	Ref
Yes	11/30	1.38 (0.67-2.84)	1.27 (0.60-2.70)	1.36 (0.62-2.95)	21/30	0.85 (0.48-1.51)	0.68 (0.33-1.39)	0.73 (0.34-1.54)
Psoriasis								
No	531/2137	Ref	Ref	Ref	1807/2137	Ref	Ref	Ref
Yes	55/218	0.91 (0.66-1.25)	0.87 (0.62-1.21)	0.82 (0.58-1.15)	196/218	1.08 (0.87-1.33)	1.07 (0.84-1.37)	1.04 (0.80-1.34)
Rheumatoid arthritis								
No	510/2059	Ref	Ref	Ref	1779/2059	Ref	Ref	Ref

Yes	76/296	0.91 (0.69-1.21)	0.88 (0.66-1.17)	0.88 (0.66-1.19)	224/296	0.86 (0.71-1.04)	0.86 (0.69-1.08)	0.84 (0.67-1.06)
Sjögren's syndrome								
No	575/2335	Ref	Ref	Ref	1988/2335	Ref	Ref	Ref
Yes	11/20	2.17 (1.00-4.70)	2.11 (0.95-4.70)	1.93 (0.85-4.39)	15/20	1.16 (0.57-2.33)	1.42 (0.65-3.12)	1.35 (0.59-3.07)
Systemic lupus erythematosus								
No	580/2341	Ref	Ref	Ref	1997/2341	Ref	Ref	Ref
Yes	6/14	1.43 (0.52-3.92)	1.44 (0.51-4.05)	1.39 (0.48-4.01)	6/14	0.59 (0.22-1.57)	0.52 (0.17-1.57)	0.62 (0.21-1.88)
Thyroid dysfunction								
No	491/2091	Ref	Ref	Ref	1769/2091	Ref	Ref	Ref
Yes	95/264	1.40 (1.07-1.83)	1.43 (1.08-1.88)	1.29 (0.97-1.71)	234/264	1.18 (0.97-1.43)	1.18 (0.93-1.49)	1.08 (0.85-1.38)
Vitiligo								
No	555/2318	Ref	Ref	Ref	1976/2318	Ref	Ref	Ref
Yes	31/37	3.08 (1.86-5.10)	3.32 (1.98-5.59)	3.45 (2.02-5.89)	27/37	0.90 (0.54-1.53)	0.94 (0.51-1.73)	0.94 (0.50-1.77)

AD = autoimmune disease. LADA = latent autoimmune disease in adults.

Model 1: adjusted for age, sex, and having any ADs at baseline. Model 2: adjusted for age, sex, having any ADs at baseline, education, smoking, physical activity, and body mass index (BMI). Model 3: adjusted for age, sex, having any ADs at baseline, education, smoking, physical activity, BMI, type 1 diabetes in first-degree relatives, and type 2 diabetes in first-degree relatives. For individual AD in first-degree relatives, the models were additionally mutually adjusted for each other.

ESM Table 12. Autoimmune disease in first-degree relatives and the risk of LADA ^{low} and LADA ^{high}. Odds ratios (OR) and 95% confidence intervals (CI).

	LADA ^{low}				LADA ^{high}			
	No cases/ controls	Model 1 OR (95% CI)	Model 2 OR (95% CI)	Model 3 OR (95% CI)	No cases/ controls	Model 1 OR (95% CI)	Model 2 OR (95% CI)	Model 3 OR (95% CI)
Family history of any AD								
No	190/1574	Ref	Ref	Ref	164/1574	Ref	Ref	Ref
Yes	87/781	0.93 (0.71-1.23)	0.92 (0.69-1.22)	0.85 (0.64-1.14)	134/781	1.44 (1.11-1.85)	1.47 (1.13-1.90)	1.34 (1.03-1.74)
Number of ADs in the family								
0	190/1574	Ref	Ref	Ref	164/1574	Ref	Ref	Ref
1	66/618	0.89 (0.66-1.20)	0.88 (0.64-1.20)	0.81 (0.59-1.12)	88/618	1.21 (0.91-1.61)	1.24 (0.93-1.66)	1.14 (0.85-1.53)
2+	21/163	1.12 (0.68-1.82)	1.06 (0.64-1.75)	1.00 (0.60-1.67)	46/163	2.26 (1.55-3.30)	2.32 (1.57-3.41)	2.08 (1.40-3.09)
continuous		*1.00 (0.82-1.21)*	*0.98 (0.81-1.20)*	*0.95 (0.77-1.17)*		*1.36 (1.16-1.60)*	*1.39 (1.18-1.63)*	*1.32 (1.12-1.56)*
Number of relatives with any AD								
0	190/1574	Ref	Ref	Ref	164/1574	Ref	Ref	Ref
1	72/649	0.93 (0.70-1.25)	0.92 (0.68-1.24)	0.85 (0.62-1.16)	93/649	1.24 (0.94-1.64)	1.27 (0.96-1.68)	1.15 (0.87-1.54)
2+	15/132	0.95 (0.54-1.67)	0.91 (0.51-1.64)	0.87 (0.48-1.57)	41/132	2.38 (1.59-3.58)	2.46 (1.63-3.73)	2.26 (1.48-3.46)
continuous		*0.96 (0.77-1.19)*	*0.94 (0.75-1.18)*	*0.90 (0.71-1.13)*		*1.42 (1.19-1.71)*	*1.45 (1.21-1.74)*	*1.37 (1.13-1.65)*
**Type of AD in the family**								
Coeliac disease								
No	271/2308	Ref	Ref	Ref	289/2308	Ref	Ref	Ref
Yes	6/47	1.15 (0.47-2.79)	1.35 (0.54-3.39)	1.22 (0.47-3.15)	9/47	1.43 (0.67-3.05)	1.76 (0.81-3.80)	1.63 (0.74-3.59)
Inflammatory bowel disease								
No	275/2301	Ref	Ref	Ref	291/2301	Ref	Ref	Ref
Yes	2/54	0.34 (0.08-1.41)	0.35 (0.08-1.45)	0.30 (0.07-1.30)	7/54	0.73 (0.31-1.69)	0.82 (0.35-1.92)	0.73 (0.31-1.73)
Multiple sclerosis								
No	276/2325	Ref	Ref	Ref	288/2325	Ref	Ref	Ref
Yes	1/30	0.30 (0.04-2.25)	0.24 (0.03-1.93)	0.24 (0.03-1.92)	10/30	2.36 (1.10-5.05)	2.29 (1.06-4.96)	2.44 (1.10-5.40)
Psoriasis								
No	257/2137	Ref	Ref	Ref	266/2137	Ref	Ref	Ref
Yes	20/218	0.72 (0.44-1.18)	0.72 (0.44-1.19)	0.71 (0.43-1.18)	32/218	1.01 (0.67-1.51)	0.95 (0.63-1.43)	0.86 (0.56-1.32)
Rheumatoid arthritis								
No	245/2059	Ref	Ref	Ref	255/2059	Ref	Ref	Ref

Yes	32/296	0.85 (0.57-1.27)	0.80 (0.53-1.21)	0.83 (0.55-1.26)	43/296	0.98 (0.68-1.42)	0.97 (0.66-1.41)	0.95 (0.65-1.38)
Sjögren's syndrome								
No	273/2335	Ref	Ref	Ref	291/2335	Ref	Ref	Ref
Yes	4/20	2.32 (0.74-7.25)	2.18 (0.65-7.31)	1.88 (0.54-6.52)	7/20	2.36 (0.95-5.89)	2.29 (0.90-5.83)	2.37 (0.92-6.10)
Systemic lupus erythematosus								
No	275/2341	Ref	Ref	Ref	294/2341	Ref	Ref	Ref
Yes	2/14	1.16 (0.24-5.57)	1.15 (0.23-5.88)	1.00 (0.19-5.12)	4/14	1.59 (0.48-5.25)	1.53 (0.44-5.25)	1.64 (0.48-5.62)
Thyroid dysfunction								
No	240/2091	Ref	Ref	Ref	242/2091	Ref	Ref	Ref
Yes	37/264	1.30 (0.88-1.91)	1.30 (0.87-1.94)	1.12 (0.74-1.70)	56/264	1.49 (1.06-2.09)	1.57 (1.11-2.22)	1.45 (1.02-2.06)
Vitiligo								
No	268/2318	Ref	Ref	Ref	276/2318	Ref	Ref	Ref
Yes	9/37	2.02 (0.93-4.38)	2.10 (0.94-4.70)	2.31 (1.02-5.25)	22/37	4.24 (2.39-7.53)	4.64 (2.59-8.31)	4.70 (2.57-8.61)

AD = autoimmune disease. LADA = latent autoimmune disease in adults.

LADA ^{low} was defined as those with glutamic acid decarboxylase antibodies (GADA) <250 U/ml, while LADA ^{high} was those with GADA ≥250 U/ml.

Model 1: adjusted for age, sex, and having any ADs at baseline. Model 2: adjusted for age, sex, having any ADs at baseline, education, smoking, physical activity, and body mass index (BMI). Model 3: adjusted for age, sex, having any ADs at baseline, education, smoking, physical activity, BMI, type 1 diabetes in first-degree relatives, and type 2 diabetes in first-degree relatives. For individual AD in first-degree relatives, the models were additionally mutually adjusted for each other.

ESM Table 13. Multiple testing correction for ADs in first-degree relatives and risk of LADA, LADA ^{high}, LADA ^{low}, and type 2 diabetes

	**LADA**		**LADA ^{high}**		**LADA ^{low}**		**Type 2 diabetes**	
	Original P-value	Adjusted P-value	Original P-value	Adjusted P-value	Original P-value	Adjusted P-value	Original P-value	Adjusted P-value
Coeliac disease	0.280390	1	0.228800	1	0.683450	1	0.740900	1
Inflammatory bowel disease	0.125880	1	0.468500	1	0.107660	0.968940	0.134600	1
Multiple sclerosis	0.440680	1	0.028100	0.252900	0.178030	1	0.402800	1
Psoriasis	0.248510	1	0.484500	1	0.188400	1	0.791000	1
Rheumatoid arthritis	0.414730	1	0.779200	1	0.389360	1	0.141000	1
Sjögren's syndrome	0.114690	1	0.073400	0.660600	0.319460	1	0.473900	1
Systemic lupus erythematosus	0.547280	1	0.429300	1	0.996110	1	0.400600	1
Thyroid dysfunction	0.082390	0.741510	0.038100	0.342900	0.580930	1	0.521300	1
Vitiligo	0.000005	0.000049	0.000001	0.000005	0.045880	0.412920	0.848500	1

AD = autoimmune disease. LADA = latent autoimmune diabetes in adults.

LADA ^{low} was defined as those with glutamic acid decarboxylase antibodies (GADA) <250 U/ml, while LADA ^{high} was those with GADA ≥250 U/ml.

The original P-values were derived from models adjusted for age, sex, having any ADs at baseline, education, smoking, physical activity, body mass index, type 1 diabetes in first-degree relatives, type 2 diabetes in first-degree relatives, and individual ADs in first-degree relatives mutually adjusted for each other. Adjusted P-values were obtained by multiplying the original P-values by nine.

ESM Table 14. Interaction between having any autoimmune disease (AD) and first-degree relatives with AD on the risk of LADA and type 2 diabetes

	No AD		Any AD	
	No cases / control	OR (95% CI)	No cases / control	OR (95% CI)
**LADA**				
Family history of AD (no)	279/1308	Ref	82/266	1.60 (1.19-2.16)
Family history of AD (yes)	130/553	1.07 (0.84-1.37)	95/228	1.88 (1.40-2.54)
Measure of interaction on additive scale: AP (95% CI) = 0.11 (-0.23; 0.46)				
**Type 2 diabetes**				
Family history of AD (no)	1107/1308	1.12 (0.92-1.35)	260/266	1.26 (0.96-1.64)
Family history of AD (yes)	417/553	Ref	219/228	1.18 (0.89-1.58)
Measure of interaction on additive scale: AP (95% CI) = -0.03 (-0.38; 0.31)				
**LADA ^{low}**				
Family history of AD (no)	154/1308	1.21 (0.85-1.71)	36/266	1.72 (1.07-2.78)
Family history of AD (yes)	53/553	Ref	34/228	1.68 (1.02-2.74)
Measure of interaction on additive scale: AP (95% CI) = -0.09 (-0.68; 0.49)				
**LADA ^{high}**				
Family history of AD (no)	120/1308	Ref	44/266	1.89 (1.27-2.79)
Family history of AD (yes)	75/553	1.36 (0.99-1.88)	59/228	2.60 (1.80-3.76)
Measure of interaction on additive scale: AP (95% CI) = 0.13 (-0.25; 0.52)				

AD = autoimmune disease. LADA = latent autoimmune diabetes in adults. AP = attributable proportion due to interaction.

LADA ^{low} was defined as those with glutamic acid decarboxylase antibodies (GADA) <250 U/ml, while LADA ^{high} was those with GADA ≥250 U/ml.

ORs were adjusted for age, sex, education, smoking, physical activity, body mass index, type 1 diabetes in first-degree relatives, and type 2 diabetes in first-degree relatives.

For type 2 diabetes and LADA ^{low}, APs were calculated by recoding the preventive factor, assigning stratum with the lowest category as the reference.

ESM Table 15. Characteristics of individuals with LADA and type 2 diabetes by autoimmune disease status at baseline

	LADA			Type 2 diabetes		
	No AD (N=388)	AD (N=198)	P-value	No AD (N=1486)	AD (N=517)	P-value
Age, mean (SD)	57.9 ± 12.4	61.2 ± 12.0	0.002	62.5 ± 10.3	64.5 ± 10.6	<0.001
Female, n (%)	156 (40.2)	118 (59.6)	<0.001	491 (33.0)	301 (58.2)	<0.001
Family history of diabetes, n (%)	241 (62.1)	132 (66.7)	0.278	974 (65.5)	332 (64.2)	0.585
Family history of type 1 diabetes, n (%)	37 (9.5)	29 (14.6)	0.064	82 (5.5)	29 (5.6)	0.938
Family history of type 2 diabetes, n (%)	180 (46.4)	100 (50.5)	0.346	846 (56.9)	282 (54.5)	0.346
Family history of any AD, n (%)	191 (49.2)	130 (65.7)	<0.001	608 (40.9)	302 (58.4)	<0.001
GADA (median; Q1, Q3)	204 (29.0, 250.0)	250.0 (50.5, 250.0)	0.037	1.0 (1.0, 1.0)	1.0 (1.0, 1.0)	0.575
C-peptide (median; Q1, Q3)	0.7 (0.5, 1.2)	0.7 (0.4, 1.2)	0.911	1.2 (1.0, 1.5)	1.3 (1.0, 1.6)	0.076
HOMA-B (median; Q1, Q3)	37.8 (13.1, 67. 8)	41.5 (16.6, 74.7)	0.650	68.9 (42.2, 94.0)	77.4 (53.2, 100.1)	<0.001
HOMA-IR (median; Q1, Q3)	2.8 (1.9, 4.4)	3.0 (1.8, 4.6)	0.795	3.6 (2.7, 4.8)	3.6 (2.8, 4.9)	0.759
HbA1c (mmol/mol) (median; Q1, Q3)	58.0 (47.0, 85.5)	57.0 (48.0, 77.0)	0.407	50.0 (44.0, 65.0)	50.0 (44.0, 58.8)	0.732
HbA1c (%) (median; Q1, Q3)	7.5 (6.5, 10.0)	7.4 (6.5, 9.2)	0.407	6.7 (6.2, 8.1)	6.7 (6.2, 7.5)	0.732
High risk – HLA genotypes, n (%)	170 (61.8)	68 (55.7)	0.254	285 (30.3)	110 (34.1)	0.203
BMI, mean (SD)	28.6 (5.6)	28.0 (5.7)	0.204	31.0 (5.2)	31.5 (5.7)	0.092
BMI ≥25 kg/m^2, n (%)	288 (74.2)	129 (65.2)	0.022	1375 (92.5)	478 (92.5)	0.956
Tertiary education, n (%)	107 (27.6)	56 (28.3)	0.805	306 (20.6)	103 (19.9)	0.402
Current smokers, n (%)	92 (23.7)	42 (21.2)	0.073	294 (19.8)	100 (19.3)	0.707
Insulin therapy, n (%)	19 (4.9)	5 (2.5)	0.171	13 (0.9)	8 (1.5)	0.196
Other glucose lowering drugs, n (%)	70 (18.0)	26 (13.1)	0.129	238 (16.0)	73 (14.1)	0.305
Insulin and other glucose lowering drugs, n (%)	85 (21.9)	30 (15.2)	0.051	244 (16.4)	76 (14.7)	0.358
Without any glucose lowering treatments, n (%)	303 (78.1)	168 (84.8)		1242 (83.6)	441 (85.3)	
Cardiovascular diseases, n (%)	42 (10.8)	22 (11.1)	0.916	259 (17.4)	103 (19.9)	0.204
Diabetic retinopathy, n (%)	5 (1.3)	8 (4.0)	0.040	37 (2.5)	9 (1.7)	0.327

BMI = body mass index. AD = autoimmune disease. LADA = latent autoimmune diabetes in adults. GADA = glutamic acid decarboxylase antibodies. HOMA – B = the Homeostasis Model Assessment of beta cell function. HOMA – IR = the Homeostasis Model Assessment of Insulin Resistance. HbA1c = glycated haemoglobin.
Information on diabetes medications (ATC A10) at diagnosis was retrieved from the Prescribed Drug Register.
Family history of type 1 or type 2 diabetes included parents, siblings, children, or grandparents with the condition, while family history of diabetes included other relatives.
High-risk genotypes were determined as carriers of DR3/3, DR3/4, DR4/4, or haplotypes of DR4-DQ8 or DR3-DQ2.
P-value is for comparison between LADA cases with vs. without AD, and type 2 diabetes cases with vs. without AD.

ESM Table 16. Sensitivity analyses of incidence of diabetic retinopathy in people with LADA with and without autoimmune co-morbidity, compared to type 2 diabetes, additionally adjusting for glucose-lowering drugs, statin, and antihypertensives. Hazard ratios (HRs) and 95% confidence intervals (95% CIs)

	**Number of events**	**Person-years**	**Model 1**<br>HR (95% CI)	**Model 2**<br>HR (95% CI)
**Diabetic retinopathy**				
Type 2 diabetes overall	116	10508	Ref	Ref
LADA overall	82	3209	1.77 (1.22-2.56)	1.49 (1.00-2.24)
LADA without AD	53	2160	1.65 (1.09-2.51)	1.43 (0.92-2.22)
LADA with AD	29	1049	2.01 (1.23-3.30)	1.65 (0.96-2.82)

AD = autoimmune disease. LADA = latent autoimmune diabetes in adults.
Model 1 was Cox proportional hazard regression model with attained age as the time scale, adjusted for sex, calendar year at diabetes diagnosis, diabetes duration, education, smoking, alcohol consumption (based on amount and frequency in the past year), physical activity, body mass index, glycated haemoglobin (HbA1c), blood pressure, lipids, estimated glomerular filtration rate (eGFR), statins (ATC C10AA, C10B), and antihypertensive drugs (ATC C02, C03, C04, C07, C08, C09). Model 2 was model 1, additionally adjusted for insulin and non-insulin glucose-lowering drugs (ATC A10A, A10B, A10X). The models were all estimated with non-diabetic retinopathy death as a competing event. The study population was people free of diabetic retinopathy at baseline.

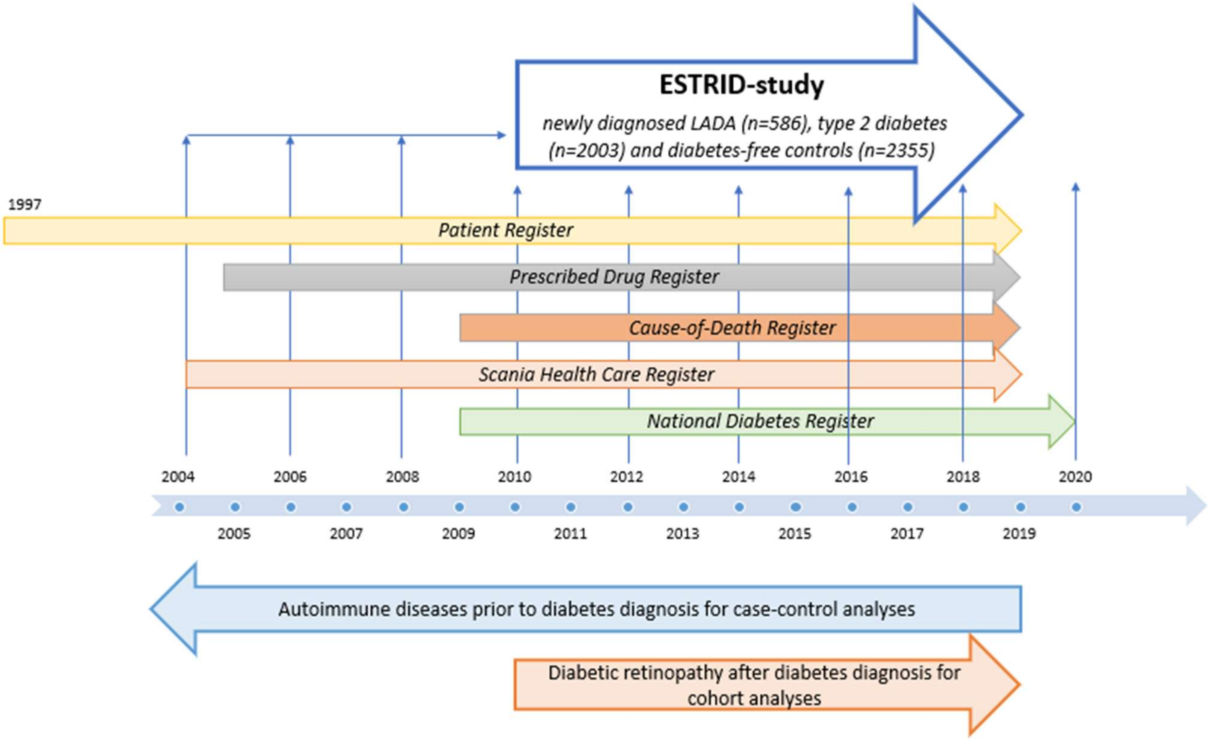

ESM Fig. 1. Study design and register linkage

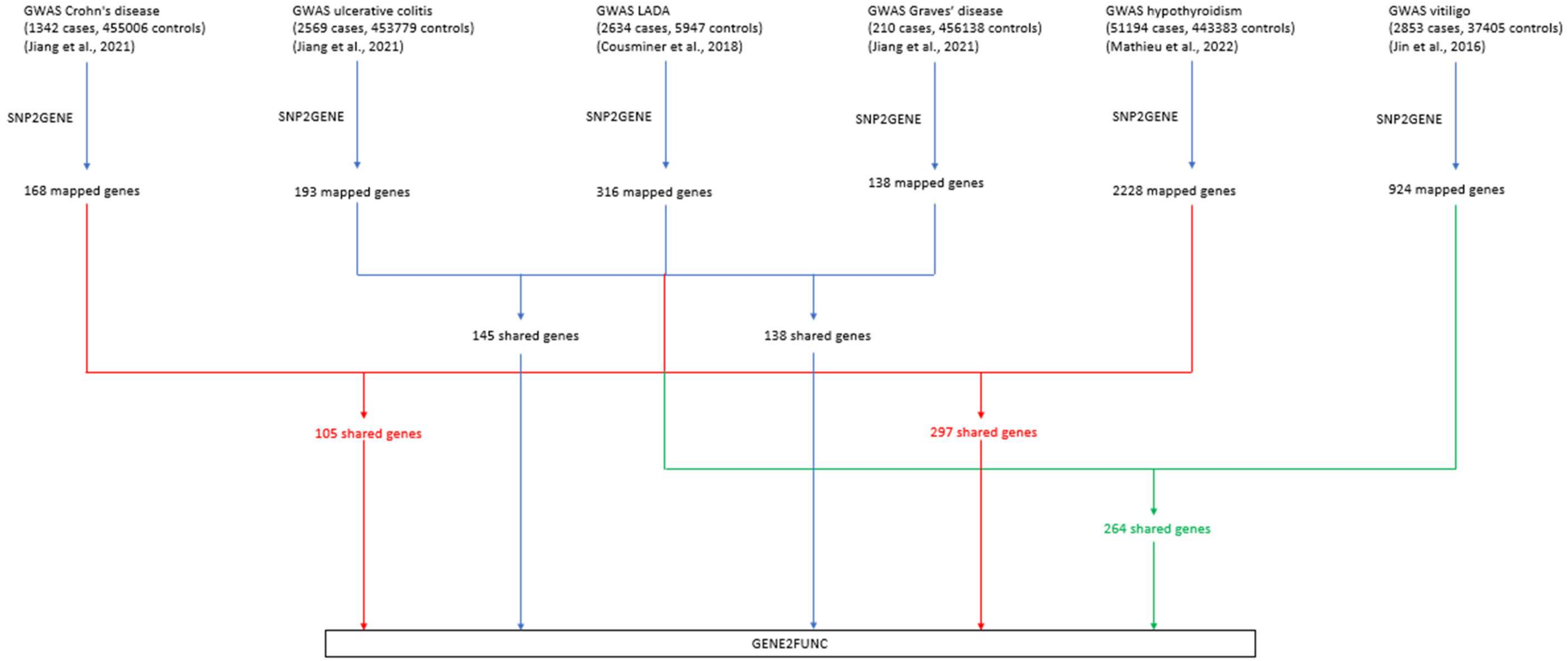

ESM Fig. 2. Flowchart of the biological pathway analyses

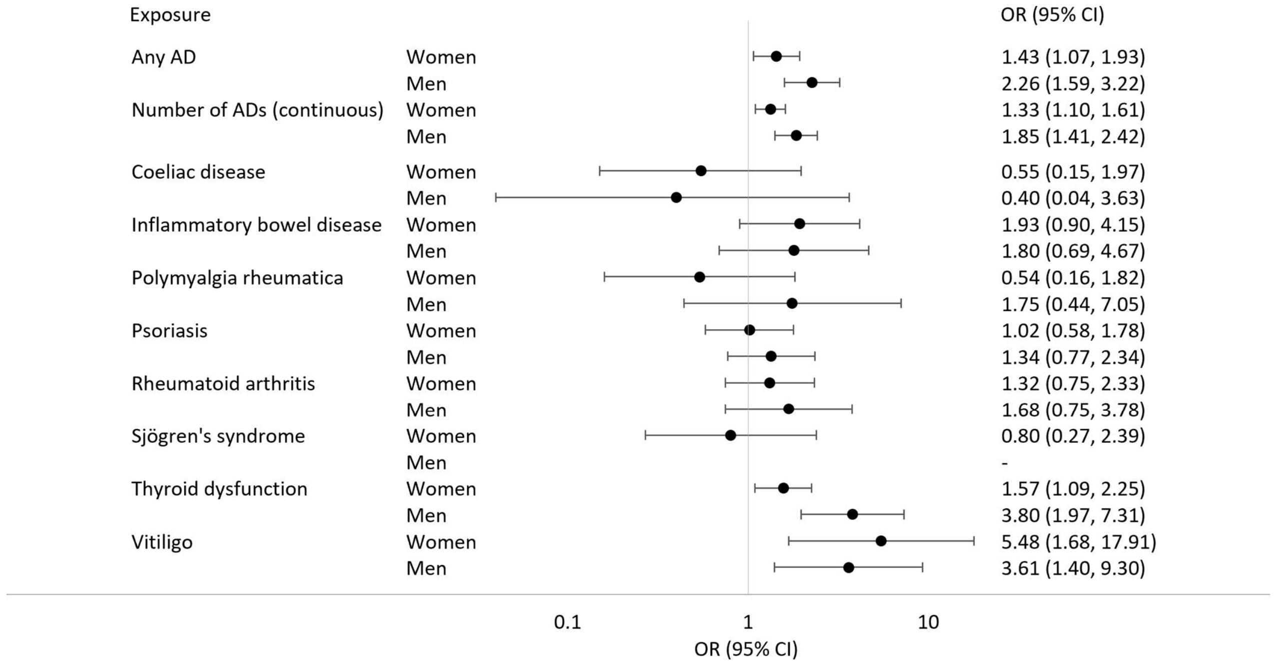

ESM Fig. 3. Autoimmune disease (AD) and risk of latent autoimmune diabetes in adults (LADA) in men and women. Odds ratios (ORs) and 95% confidence intervals (CIs) were adjusted for age, sex, education, smoking, physical activity, body mass index, family history of type 1 diabetes, family history of type 2 diabetes, and family history of any AD. ORs for individual ADs were mutually adjusted for the other ADs.

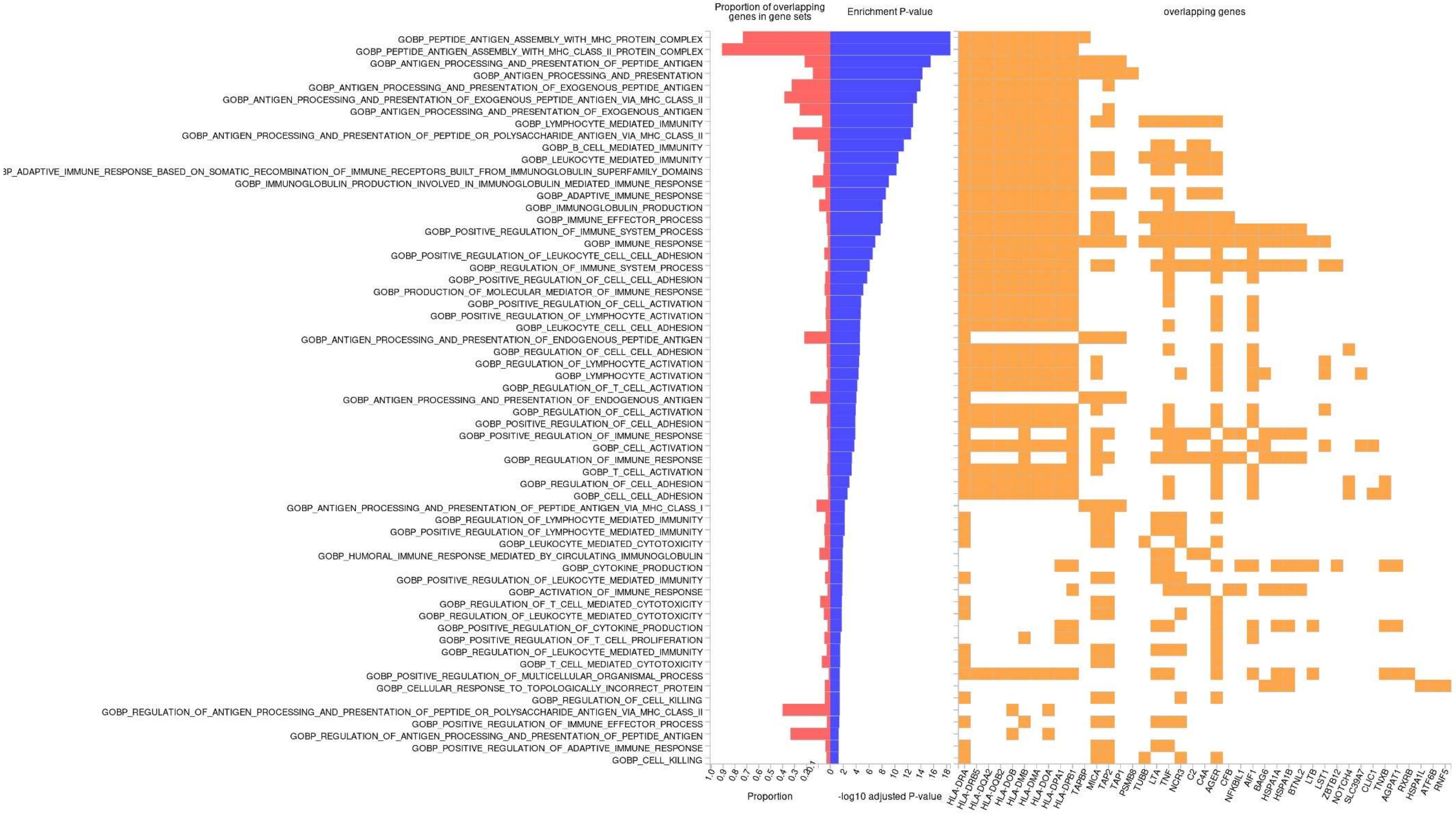

ESM Fig. 4. Biological pathways between Crohn's disease and LADA. The picture shows the Gene Ontology (GO) categories for biological processes (left), proportion of the overlapping genes (input genes compared to background gene-sets), enrichment P-value (the associated -log10 adjusted p-value after false discovery rate (FDR) correction (i.e., Benjamini–Hochberg)), and the overlapping genes associated with the biological processes (right).

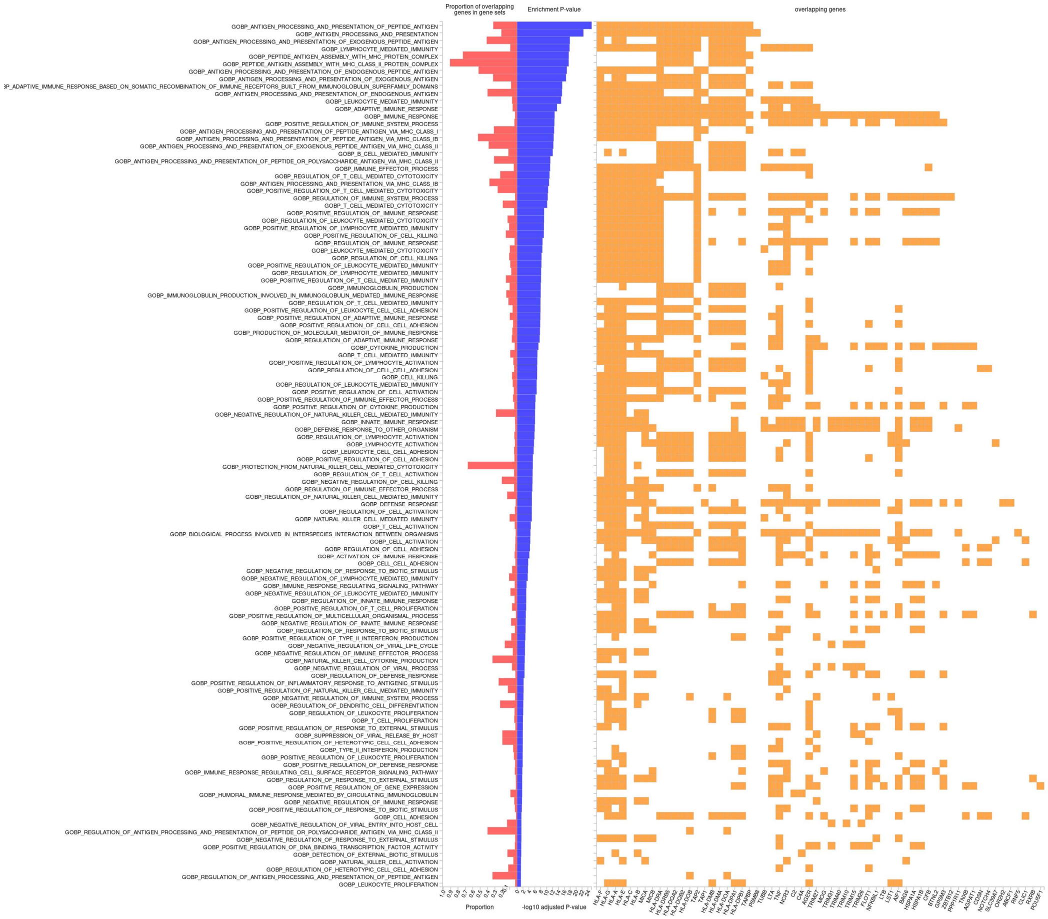

ESM Fig. 5. Biological pathways between ulcerative colitis and LADA. The picture shows the Gene Ontology (GO) categories for biological processes (left), proportion of the overlapping genes (input genes compared to background gene-sets), enrichment P-value (the associated -log10 adjusted p-value after false discovery rate (FDR) correction (i.e., Benjamini–Hochberg)), and the overlapping genes associated with the biological processes (right).

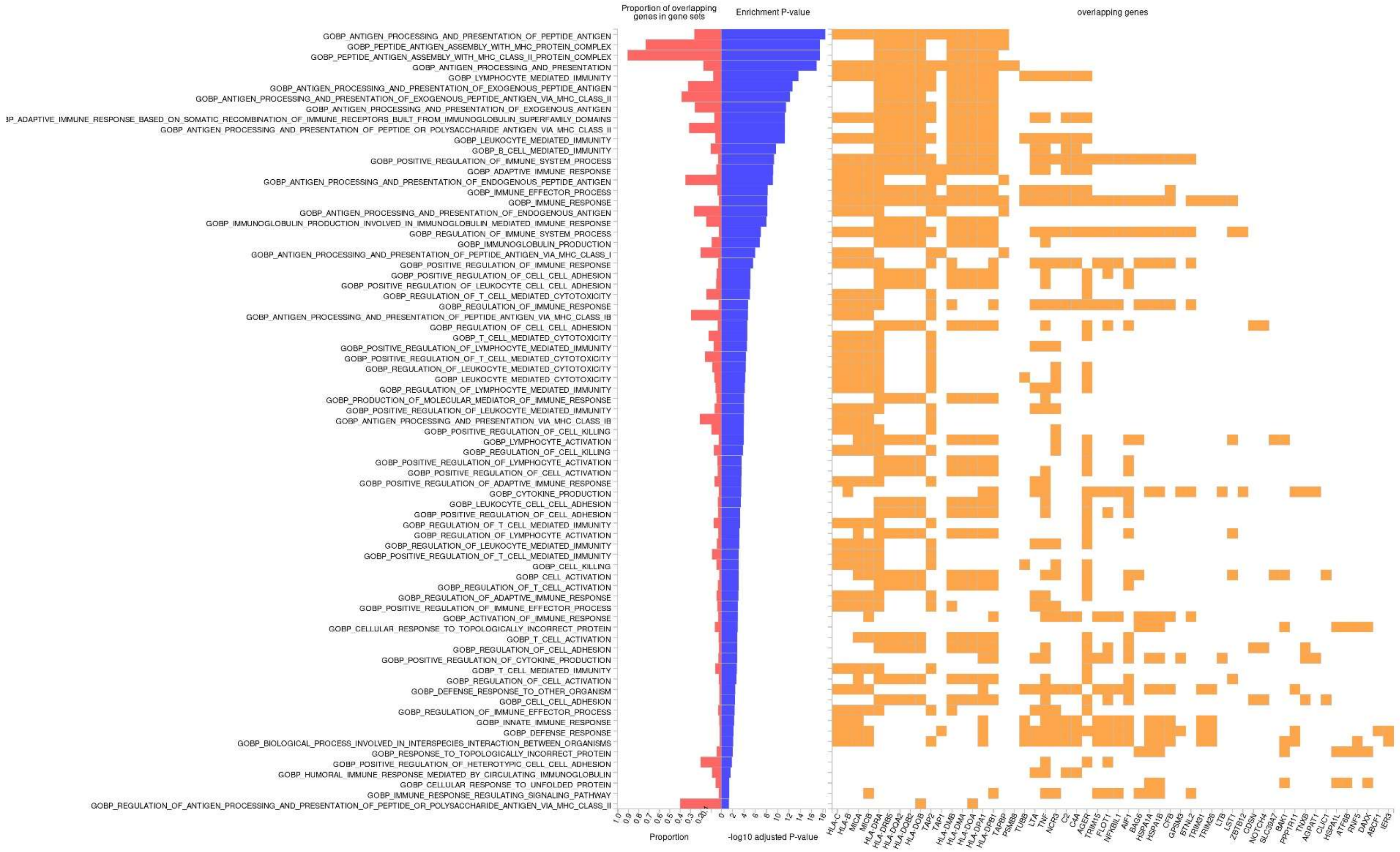

ESM Fig. 6. Biological pathways between Graves' disease and LADA. The picture shows the Gene Ontology (GO) categories for biological processes (left), proportion of the overlapping genes (input genes compared to background gene-sets), enrichment P-value (the associated -log10 adjusted p-value after false discovery rate (FDR) correction (i.e., Benjamini–Hochberg)), and the overlapping genes associated with the biological processes (right).

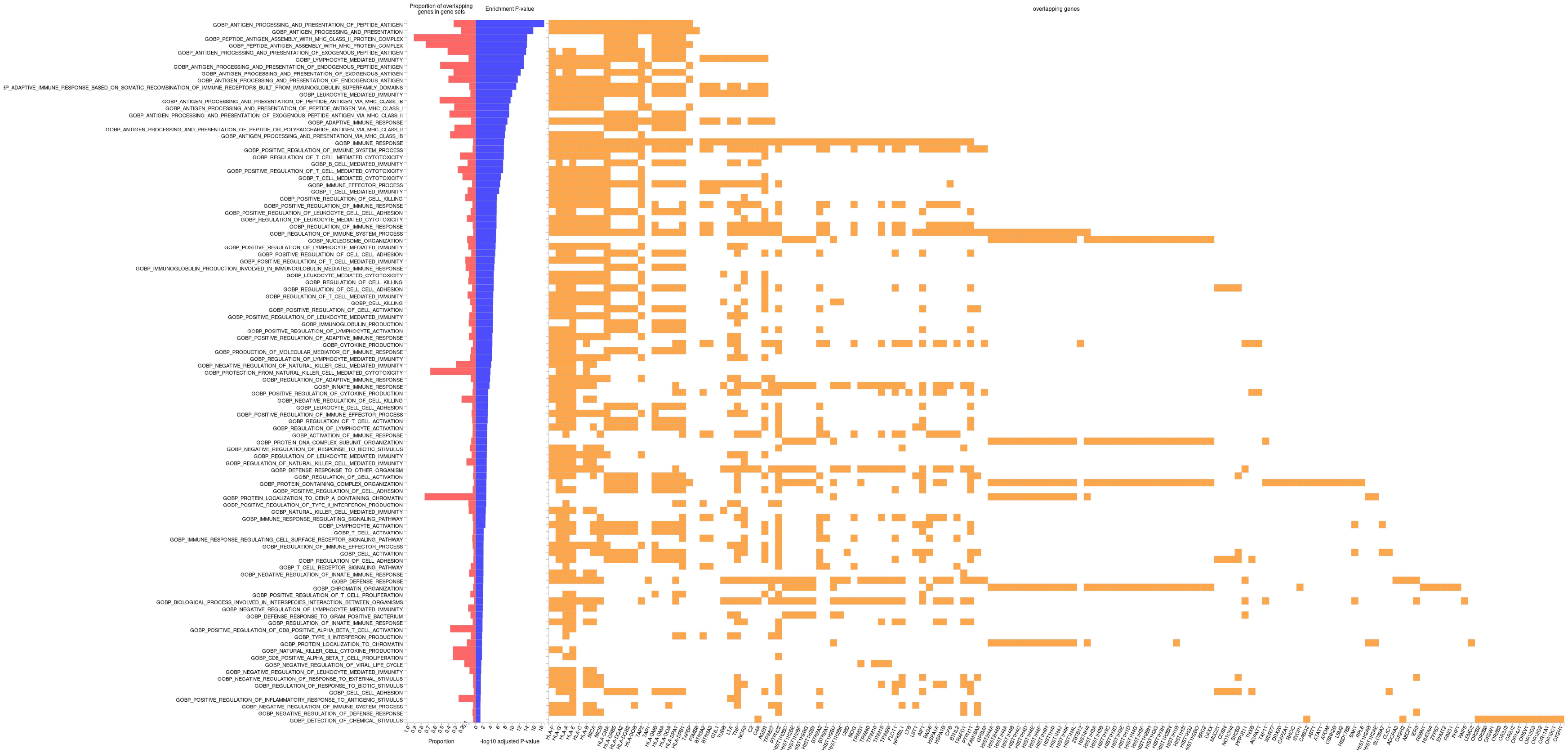

ESM Fig. 7. Biological pathways between hypothyroidism and LADA. The picture shows the Gene Ontology (GO) categories for biological processes (left), proportion of the overlapping genes (input genes compared to background gene-sets), enrichment P-value (the associated -log10 adjusted p-value after false discovery rate (FDR) correction (i.e., Benjamini–Hochberg)), and the overlapping genes associated with the biological processes (right).

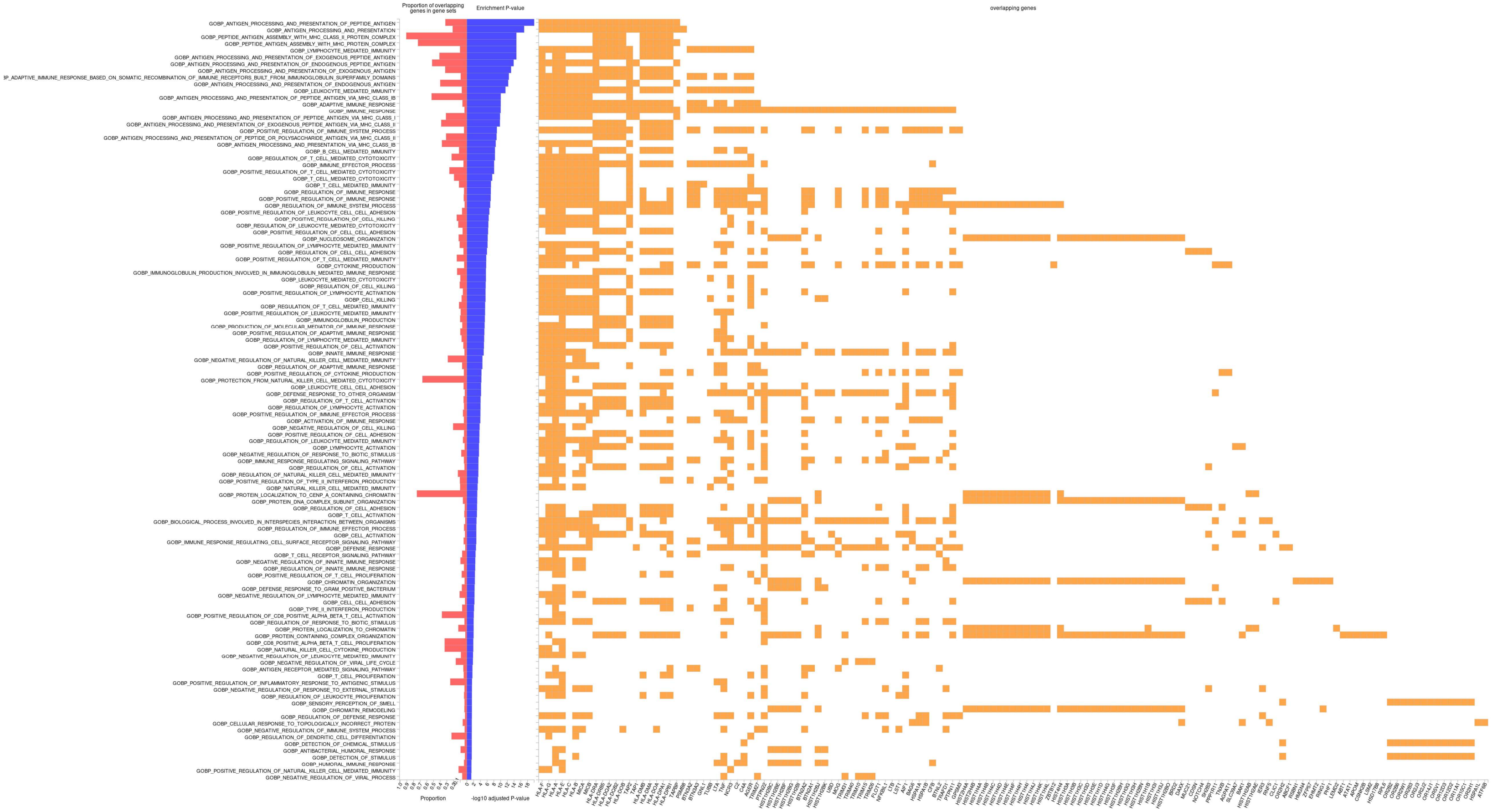

ESM Fig. 8. Biological pathways between vitiligo and LADA. The picture shows the Gene Ontology (GO) categories for biological processes (left), proportion of the overlapping genes (input genes compared to background gene-sets), enrichment P-value (the associated -log10 adjusted p-value after false discovery rate (FDR) correction (i.e., Benjamini–Hochberg)), and the overlapping genes associated with the biological processes (right).

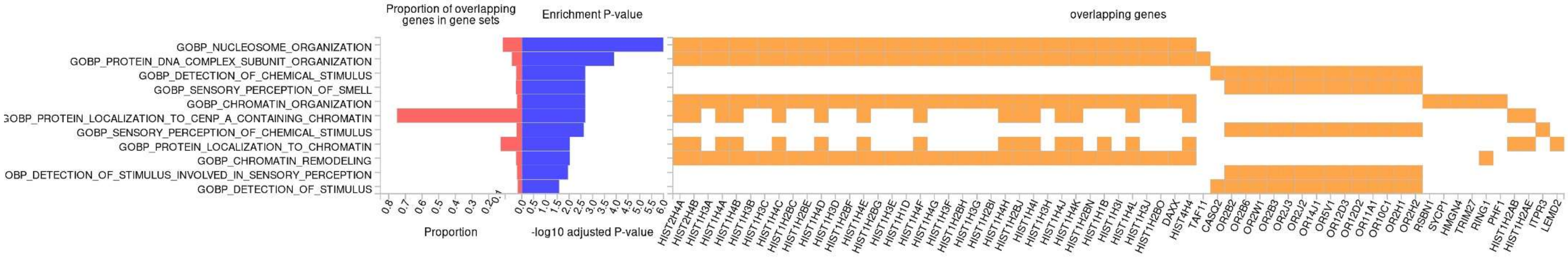

ESM Fig. 9. Biological pathways between hypothyroidism and LADA (excluding major histocompatibility complex). The picture shows the Gene Ontology (GO) categories for biological processes (left), proportion of the overlapping genes (input genes compared to background gene-sets), enrichment P-value (the associated -log10 adjusted p-value after false discovery rate (FDR) correction (i.e., Benjamini–Hochberg)), and the overlapping genes associated with the biological processes (right).

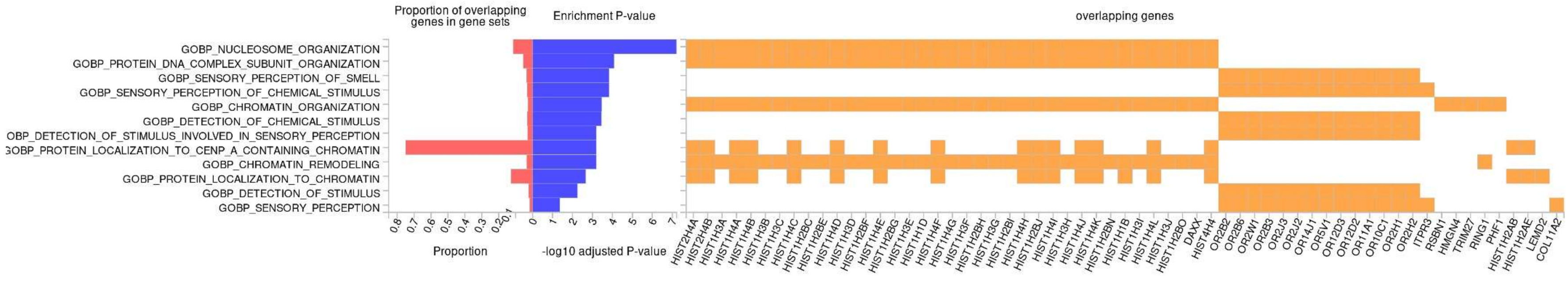

ESM Fig. 10. Biological pathways between vitiligo and LADA (excluding major histocompatibility complex). The picture shows the Gene Ontology (GO) categories for biological processes (left), proportion of the overlapping genes (input genes compared to background gene-sets), enrichment P-value (the associated -log10 adjusted p-value after false discovery rate (FDR) correction (i.e., Benjamini–Hochberg)), and the overlapping genes associated with the biological processes (right).

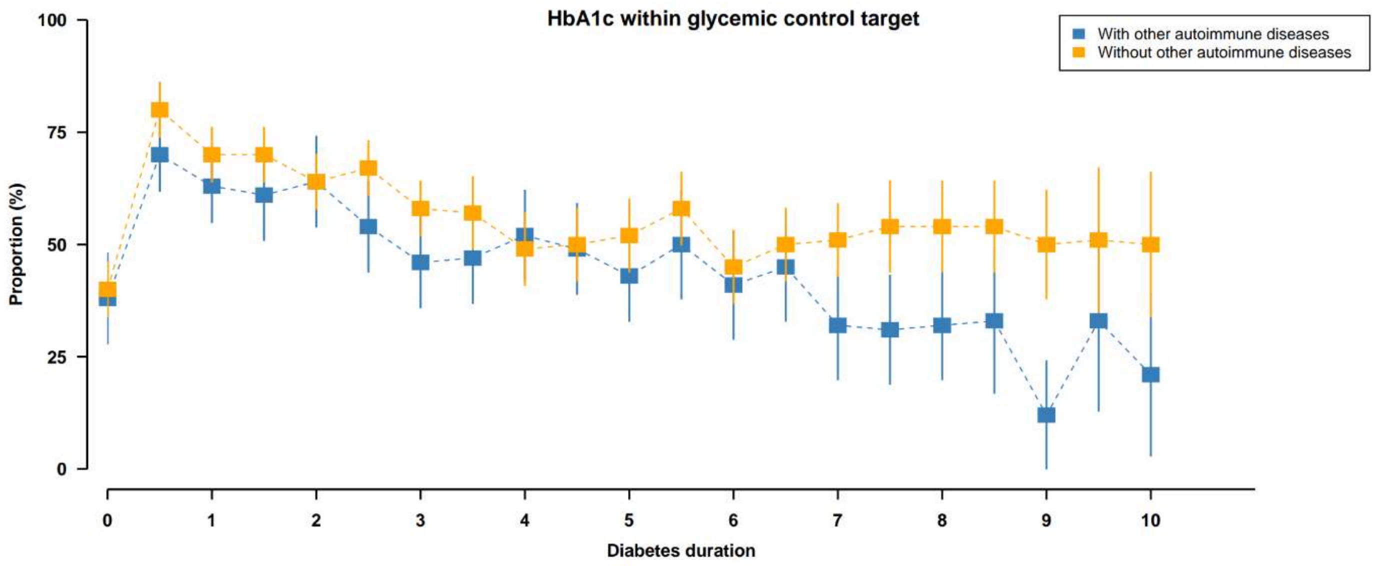

ESM Fig. 11. Long-term glycated haemoglobin (HbA1c) levels trajectories comparing individuals with latent autoimmune diabetes in adults (LADA) with and without autoimmune diseases. The trajectories were estimated by generalized linear models with cluster robust standard errors. HbA1c <53 mmol/mol was considered as within control target.
